# SEED-Selection enables high-efficiency enrichment of primary T cells edited at multiple loci

**DOI:** 10.1038/s41587-024-02531-6

**Published:** 2025-02-05

**Authors:** Christopher R. Chang, Vivasvan S. Vykunta, Jae Hyun J. Lee, Ke Li, Clara Kochendoerfer, Joseph J. Muldoon, Charlotte H. Wang, Thomas Mazumder, Yang Sun, Daniel B. Goodman, William A. Nyberg, Chang Liu, Vincent Allain, Allison Rothrock, Chun J. Ye, Alexander Marson, Brian R. Shy, Justin Eyquem

**Affiliations:** 1 Gladstone-UCSF Institute of Genomic Immunology, San Francisco, CA USA; 2 Department of Medicine, University of California, San Francisco, San Francisco, CA USA; 3 Medical Scientist Training Program, University of California, San Francisco, San Francisco, CA USA; 4 Biomedical Sciences Graduate Program, University of California, San Francisco, San Francisco, CA USA; 5 Department of Laboratory Medicine, University of California, San Francisco, San Francisco, CA USA; 6 Parker Institute for Cancer Immunotherapy, San Francisco, CA USA; 7 Division of Rheumatology, University of California, San Francisco, San Francisco, CA USA; 8 Diabetes Center, University of California, San Francisco, San Francisco, CA USA; 9 Université Paris Cité, INSERM UMR976, Hôpital Saint-Louis, Paris, France; 10 Department of Microbiology and Immunology, University of California, San Francisco, San Francisco, CA USA; 11 Institute for Human Genetics, University of California, San Francisco, San Francisco, CA USA; 12 Bakar Computational Health Sciences Institute, University of California, San Francisco, San Francisco, CA USA; 13 Department of Epidemiology and Biostatistics, University of California, San Francisco, San Francisco, CA USA; 14 Arc Institute, Palo Alto, CA USA; 15 Department of Bioengineering and Therapeutic Sciences, University of California, San Francisco, San Francisco, CA USA; 16 Bakar Aging Research Institute, University of California, San Francisco, San Francisco, CA USA; 17 Innovative Genomics Institute, University of California, Berkeley, Berkeley, CA USA; 18 UCSF Helen Diller Family Comprehensive Cancer Center, University of California, San Francisco, San Francisco, CA USA

**Keywords:** Cell therapies, Genetic engineering, Synthetic biology, Cancer immunotherapy

## Abstract

Engineering T cell specificity and function at multiple loci can generate more effective cellular therapies, but current manufacturing methods produce heterogenous mixtures of partially engineered cells. Here we develop a one-step process to enrich unlabeled cells containing knock-ins at multiple target loci using a family of repair templates named synthetic exon expression disruptors (SEEDs). SEEDs associate transgene integration with the disruption of a paired target endogenous surface protein while preserving target expression in nonmodified and partially edited cells to enable their removal (SEED-Selection). We design SEEDs to modify three critical loci encoding T cell specificity, coreceptor expression and major histocompatibility complex expression. The results demonstrate up to 98% purity after selection for individual modifications and up to 90% purity for six simultaneous edits (three knock-ins and three knockouts). This method is compatible with existing clinical manufacturing workflows and can be readily adapted to other loci to facilitate production of complex gene-edited cell therapies.

## Abstract (web summary)

Primary T cells with multiple genetic modifications are rapidly isolated by negative selection.

## Main

T cells engineered to express synthetic immune receptors are highly effective for the treatment of refractory hematological malignancies^1,2^. Nevertheless, efforts to create new cell therapies have been stymied by difficulties maintaining T cell persistence and functionality^3,4^. Furthermore, widespread adoption of cell therapies has been hindered by the autologous nature of current products, which require an expensive and time-consuming individualized manufacturing process^5^.

Combinations of transgenes integrated into the genome through CRISPR–Cas editing have been used to improve the performance of cell therapies or to create allogeneic (off-the-shelf) products^6–9^. However, viral and nonviral DNA repair templates have a limited cargo capacity, which constrains the number and size of transgenes that can be introduced at one locus^10,11^. CRISPR–Cas can be used to introduce targeted double-strand breaks (DSBs) at multiple loci simultaneously but achieving multiple transgene integrations is challenging because nonhomologous end joining (NHEJ), which produces insertions and deletions (indels), generally outcompetes homology-directed repair (HDR)-mediated transgene integration^12^. As a result, efforts to perform multiplexed knock-ins have yielded numerous populations of partially edited cells that can perform suboptimally^13–16^. Because product purity is critical for clinical manufacturing, efficient methods for isolating fully edited cells are necessary for the realization of multilocus integration strategies.

Isolating engineered cells has been a long-lasting interest of the field and a variety of methods have been developed. Surface tags and drug resistance cassettes have been used to enrich for cells with transgene integrations but subjecting cells to multiple drugs or performing sequential rounds of positive selection can negatively impact cell viability, performance and yield^16–19^. Alternatively, the targeting of essential loci has been used to enrich for cells with transgene integrations^20^; however, the consequences of simultaneously editing multiple essential genes have not yet been evaluated. As editing outcomes at distinct loci are linked, previous studies used a selective marker introduced at one locus to enrich for integrations at another locus^15,21,22^. However, an enrichment method for direct multimarker selection would allow for the isolation of purer populations.

**Extended Data Fig. 1:**
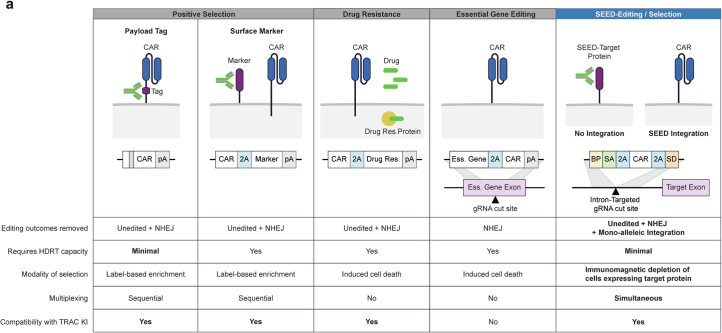
Comparison of SEED-Selection to alternative enrichment strategies. Chart detailing different strategies for isolating cells engineered to express a CAR or other transgene. Representative HDRTs are shown for each selection strategy. *: STOP codon; BP: branchpoint; SA: splice acceptor; SD: splice donor; pA: polyA signal; LHA/RHA: homology arms.

Here, we develop a one-step, drug-free process to isolate unlabeled cells that have transgene integrations at multiple loci. We devise a type of repair template named synthetic exon expression disruptor (SEED) to link successful transgene integration with the disruption of a paired endogenous surface protein, allowing cells with knock-ins to be enriched through immunomagnetic negative selection (SEED-Selection) (Extended Data Fig. 1). We design SEEDs to disrupt three translationally relevant surface proteins in primary human T cells while facilitating the expression of various therapeutic payloads. We characterize editing outcomes and transgene function in cells edited with a single or multiple SEEDs and the ability of SEED-Selection to enrich for cells with biallelic integrations in a single step. Additionally, we demonstrate that antibody epitope editing enables the enrichment of transgenes that would otherwise be depleted during this process and facilitates the removal of T cells with mispaired T cell receptors (TCRs) when a transgenic TCR is introduced at the TCRα constant (*TRAC*) locus. SEED-Selection facilitates the isolation of almost entirely pure (up to 98%) populations of cells with an intended knock-in and knockout. Furthermore, SEED-Selection is amenable to multiplexing, allowing for the isolation of highly pure (up to 90% fully edited) populations that have three knock-ins and three knockouts. SEED-Selection could be easily adapted to various cell types to facilitate clinical manufacturing of complex gene-edited cell therapies.

## Results

### SEEDs enable enrichment of cells with transgene integrations

**Fig. 1:**
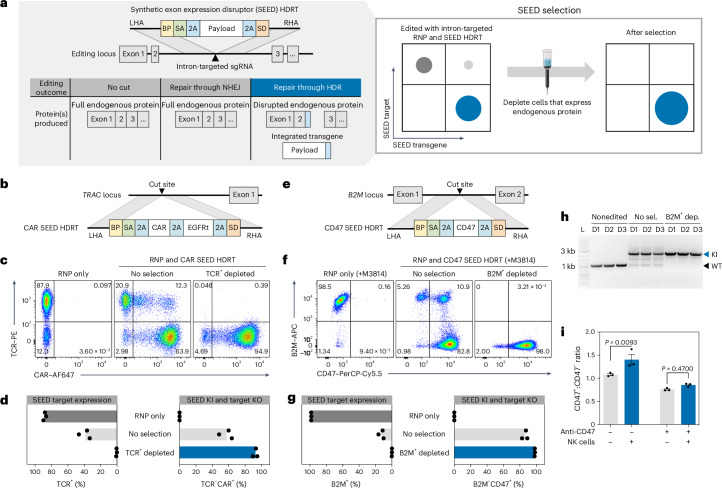
SEEDs couple transgene integration to the target protein disruption. **a**, Overview of editing outcomes generated with an intron-targeted single gRNA (sgRNA) and a SEED HDRT. Immunomagnetic reagents are used to deplete cells that retain expression of the surface protein targeted by a SEED, thereby enriching for cells with transgene integration. **b**, Diagram of a *TRAC* intron-targeted SEED HDRT encoding a CAR and EGFRt. **c**,**d**, T cells were edited with *TRAC* intron-targeted RNP and HDRT (**b**) and then immunomagnetically purified with anti-TCR (*n* = 3 donors). **c**, Flow cytometry plots of TCR and CAR expression (anti-mouse F(ab′)_2_). **d**, Percentage of TCR^+^ and TCR^−^ CAR^+^ cells. KI, knock-in; KO, knockout. **e**, Diagram of a *B2M* intron-targeted SEED HDRT encoding CD47. **f**–**h** T cells were edited with *B2M* intron-targeted RNP and HDRT (**e**) in the presence of M3814 then immunomagnetically purified with anti-B2M (*n* = 3 donors). **f**, Flow cytometry plots of B2M and CD47 expression. **g**, Percentage of B2M^+^ and B2M^–^ CD47^+^ cells. **h**, Genomic DNA PCR targeting the SEED integration site at *B2M*. Amplicon for nonedited alleles, black triangle; amplicon for HDRT integration, blue triangle (*n* = 3 donors). No sel.; no selection; B2M^+^ dep.; B2M^+^ depleted. **i**, Fold enrichment of CD47^+^ SEED-edited cells relative to CD47^−^ cells after coculture with NK cells (*n* = 3 T cell donors). Data are displayed as the mean ± s.e.m. Significance was assessed with a two-way analysis of variance (ANOVA) and Šidák’s multiple-comparisons test. LHA, left homology arm; RHA, right homology arms. APC, allophycocyanin; PE, phycoerythrin. Source data

To develop a method for cells with transgene integrations to be enriched through negative selection, we designed two reagents: (1) a guide RNA (gRNA) targeting an intron of a surface-expressed protein that generates a DSB that minimally impacts expression and (2) a SEED HDR template (HDRT) that uses synthetic splice acceptor (SA) and splice donor (SD) sequences to introduce an in-frame transgene payload preceded by a P2A sequence at a position that disrupts the target protein (Fig. 1a).

**Extended Data Fig. 2:**
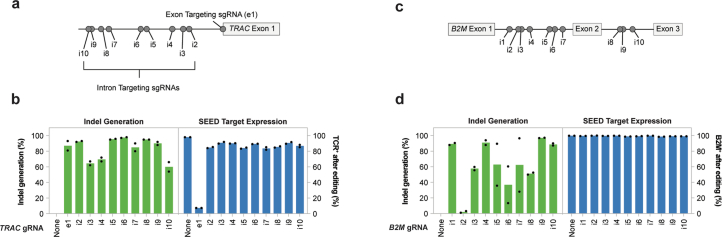
Intronic editing sites preserve target gene expression. **a**, Panel of gRNAs targeting TRAC exon (e1) and TRAC intron (i2–i10); not depicted to scale. **b**, Assessment of TCR expression by flow cytometry and indel generation by bulk genomic DNA sequencing in T cells after editing with gRNAs depicted in b (n = 2 donors). **c**, Panel of gRNAs targeting B2M introns; not depicted to scale. **d**, Assessment of B2M expression by flow cytometry and indel generation by bulk genomic DNA sequencing in T cells after editing with gRNAs depicted in c (n = 2 donors). Source data

We initially targeted the two most common loci for therapeutic T cell engineering, *TRAC* and *B2M* (β2 microglobulin). Insertion of a chimeric antigen receptor (CAR) or transgenic TCR at the *TRAC* locus can redirect T cell specificity and benefits from endogenous regulatory elements to produce potent and durable T cell therapies^23,24^. B2M is required for major histocompatibility complex class I (MHC-I) expression and can be disrupted to help evade host T cell recognition in allogeneic settings^25^. To identify optimal SEED integration sites for *TRAC* and *B2M*, we designed two panels of intron-targeted gRNAs and screened for guides that generate indels in primary human T cells without disrupting TCR or B2M surface expression, respectively (Extended Data Fig. 2a–d). Candidate gRNAs exhibited high indel generation efficiencies (up to 97%) with minimal disruption of TCR (9–16%) or B2M (1–2%) surface expression.

**Extended Data Fig. 3:**
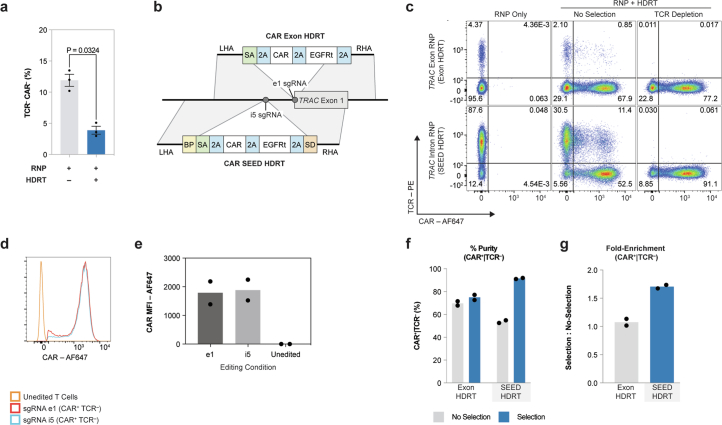
Evaluation of TRAC intron and exon targeted gRNAs. **a**, Percentage of TCR– CAR– cells after editing with TRAC RNP alone or RNP with CAR HDRT (n = 3 donors). Data displayed as mean plus SEM. Significance was assessed with a two-sided paired t test. **b**, Comparison of TRAC-intron and exon-targeted HDRTs encoding a CAR and EGFRt. **c**–**g**, T cells were edited with TRAC intron or TRAC exon-targeted RNPs and HDRTs (**b**). Edited cells from each condition were then immunomagnetically purified with anti-TCR (n = 2 donors). **c**, Flow cytometry plots of TCR and CAR (anti-mouse F(ab’)2) expression **d**, Histograms of CAR expression in TCR– CAR+ cells. **e**, Median fluorescence intensity (MFI) of CAR expression in TCR– CAR+ cells. **f**, Percentage of TCR– CAR+ cells in purified (blue) and non-purified samples (grey). **g**, Relative enrichment of fully edited cells after purification: (% of purified sample) / (% of non-purified sample). Source data

**Extended Data Fig. 4:**
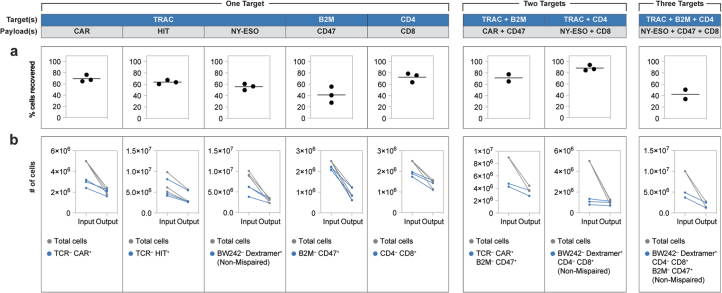
Recovery of edited cells following SEED-Selection. **a**, Estimated percentage of target cell population recovered following SEED-Selection. **b**, Input and output of total cells and target cells for SEED-Selection. Purifications for each target-payload purification were performed separately. Dots represent data from biological replicates within an experiment (n = 2 donors for TRAC-CAR/B2M-CD47 double KI purification and TRAC-NY-ESO/B2M-CD47/CD4-CD8 triple KI purification; n = 3 donors for all other conditions) Additional yield data is included in Supplementary Table 1. Source data

To confirm that SEED integration could disrupt TCR surface expression, we edited T cells with a *TRAC* intron-targeted ribonucleoprotein (RNP) and an adeno-associated viral vector (AAV6) encoding a SEED HDRT for a CD19-specific CAR and a truncated epidermal growth factor receptor (EGFRt) tag (Fig. 1b,c)^18^. Integration-mediated TCR disruption (TCR^−^CAR^+^) occurred in over half (57.4%) of SEED-transduced cells. SEED-transduced cells also exhibited less integration-independent TCR disruption (TCR^−^CAR^−^) than cells edited with the RNP alone, in agreement with reports that template-mediated HDR can outcompete pathways that create large deletions (Fig. 1c and Extended Data Fig. 3a)^26^. Although most T cells express one TCRα chain, a subset of TCR^+^CAR^+^ cells was also observed (Fig. 1c), which could result from monoallelic HDRT integration in the ~30% of T cells that exhibit transcription at both TCRα alleles^27^. Immunomagnetic TCR depletion removed nearly all TCR^+^ cells and recovered a highly pure (92.6%) TCR^−^CAR^+^ population with yields of up to 76% (Fig. 1c,d and Extended Data Fig. 4).

To compare editing outcomes between SEED and exon-targeting strategies, we designed and tested an analogous CAR HDRT with an integration site in *TRAC* exon 1 (Extended Data Fig. 3b,c)^18^. CAR expression and homogeneity were similar in T cells edited with both constructs (Extended Data Fig. 3d,e). However, immunomagnetic TCR depletion of cells edited with an exon-targeted HDRT minimally enriched for TCR^−^CAR^+^ cells, as the majority of CAR^−^ cells were also TCR^−^ (Extended Data Fig. 3f,g). These data demonstrate that SEEDs enable edited cells to be enriched by coupling transgene integration to target protein disruption.

### NHEJ inhibitors enable efficient biallelic SEED integration

The TCR is subject to multiple pretranslational and posttranslational processes that result in functional allelic exclusion and editing at the single active TCRα allele is often sufficient to ablate TCR expression^23,27^. In contrast, editing at both alleles is necessary to fully disrupt B2M and most other loci. To identify the editing conditions necessary for biallelic SEED integration, we tested an AAV6 SEED HDRT designed to disrupt B2M expression and deliver CD47, an immune checkpoint molecule that has been shown to inhibit natural killer (NK) and macrophage activity against cells lacking MHC-I expression (Fig. 1e)^28–30^.

**Extended Data Fig. 5:**
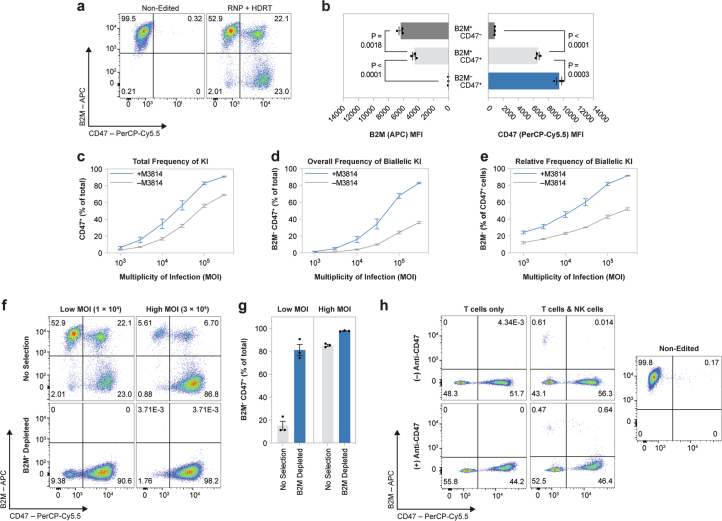
Optimization and characterization of *B2M* targeted SEEDs. **a**–**e**, T cells were edited with RNP targeting B2M and transduced with varying MOIs of AAV encoding CD47. In indicated conditions, editing was performed in the presence of M3814 (n = 3 donors). Cells transduced at an MOI of 1 × 104 and edited in the presence of M3814 shown in select panels (**a**,**b**). **a**, Representative flow cytometry plots of B2M and CD47 expression in edited and non-edited cells. **b**, Median fluorescence intensity (MFI) of B2M or CD47 expression in subpopulations of edited cells. Data displayed as mean plus SEM. Significance was assessed using a repeated-measures one-way ANOVA and Turkey’s multiple comparison test. **c**, Percentage of CD47+ cells, d, Percentage of B2M– CD47+ cells, e, Percentage of CD47+ cells with full B2M disruption. Data for c–e displayed as mean plus SEM. **f**,**g**, T cells edited with RNP targeting B2M ( + M3814) and transduced with high (3 × 105) or low (1 × 104) MOIs of B2M-CD47 SEED HDRT AAV were immunomagnetically purified with anti-B2M (n = 3 donors). **f**, Representative flow cytometry plots of B2M and CD47 expression. **g**, Percentage of B2M– CD47+ cells. Data displayed as mean plus SEM (n = 3 donors). **h**, Representative flow cytometry plots of B2M and CD47 expression used to evaluate CD47 + :CD47 ratio in Fig. 1h. Source data

We characterized editing outcomes in T cells transduced at multiplicities of infection (MOIs) ranging from 1 × 10^3^ to 3 × 10^5^ (Extended Data Fig. 5a–e). In a subset of conditions, editing was performed in the presence of M3814, an NHEJ inhibitor that promotes biallelic transgene integration^31^. After editing, distinct B2M^+^CD47^+^ and B2M^−^CD47^+^ populations were observed (Extended Data Fig. 5a). B2M^+^CD47^+^ cells expressed intermediate levels of B2M and CD47 (corresponding to monoallelic SEED integration), while B2M^−^CD47^+^ cells exhibited loss of B2M expression and increased CD47 expression (corresponding to biallelic SEED integration) (Extended Data Fig. 5a,b).

Biallelic SEED integration was achieved in 83% of cells following transduction at the highest MOI and with M3814 (Extended Data Fig. 5d). As expected, biallelic SEED integration was reduced when lower MOIs were used or when editing was performed without M3814 (Extended Data Fig. 5d,e)^31^. However, biallelic integration was still detectable in the least efficient editing conditions (lowest MOI), where the total fraction of CD47^+^ cells was <10% (Fig. 5c–e). These data suggest that optimized editing conditions enable biallelic integration to become the primary editing outcome but are not required for biallelic integration to occur.

### SEED-Selection isolates cells with biallelic integrations

To test whether SEED-Selection could enable the isolation of cells with biallelic transgene integrations, we performed immunomagnetic B2M depletion after editing cells with a *B2M*-targeted SEED HDRT (Fig. 1f,g). Immunomagnetic selection efficiently depleted B2M^+^CD47^−^ and B2M^+^CD47^+^ cells and enabled the recovery of a highly pure (>98%) population of cells with biallelic SEED integration (B2M^−^CD47^+^), with yields of up to 56% (Fig. 1f,g and Extended Data Fig. 4). Product purity was further evaluated through genomic DNA PCR, which confirmed depletion of nonedited *B2M* alleles during selection (Fig. 1h).

To assess the performance of SEED-Selection in samples with different editing distributions, we immunomagnetically purified samples transduced with high (3 × 10^5^) or low (1 × 10^4^) MOIs (Extended Data Fig. 5f,g). While the final purity of B2M^−^CD47^+^ cells was higher in samples transduced with a high MOI, SEED-Selection resulted in a larger improvement in B2M^−^CD47^+^ purity in samples transduced with a low MOI (Extended Data Fig. 5f,g). These data demonstrate the versatility of SEED-Selection across different editing scenarios.

To test whether CD47 expression was sufficient to reduce NK cell cytotoxicity, we generated a mixture of T cells with two subsets (B2M^−^CD47^+^ and B2M^−^CD47^−^) and performed a coculture with activated primary human NK cells. The composition of the coculture was then quantified by flow cytometry and compared to that of T cells alone. As intended, B2M^−^CD47^+^ cells were enriched relative to B2M^−^CD47^−^ cells after coculture (Fig. 1i and Extended Data Fig. 5h). B2M^−^CD47^+^ enrichment was not observed when T cells were pretreated with a CD47-blocking antibody, confirming that SEED-mediated CD47 activity facilitated NK cell evasion (Fig. 1i and Extended Data Fig. 5h).

### SEED-Selection simultaneously enriches for multilocus edits

**Fig. 2:**
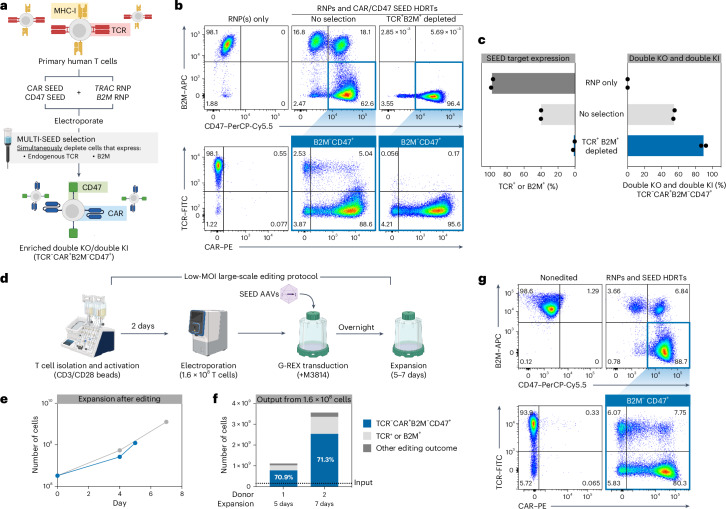
SEED-Selection can simultaneously enrich for edits at multiple loci. **a**–**c**, T cells were edited with *TRAC* and *B2M* RNPs (+M3814) and transduced with *TRAC*-CAR SEED and *B2M*-CD47 SEED HDRTs. Edited cells were then immunomagnetically purified with anti-B2M and anti-TCR (*n* = 2 donors). **a**, Multiplexed editing and enrichment strategy. **b**, Representative flow cytometry plots of TCR, CAR, B2M and CD47 expression. **c**, Percentage of B2M^+^ or TCR^+^ cells and fully edited cells (TCR^−^CAR^+^B2M^−^CD47^+^). –d–g 1.6 × 10^8^ T cells were edited with *TRAC* and *B2M* RNPs and transduced with *TRAC*-CAR SEED and *B2M*-CD47 SEED HDRTs using a GMP-compatible workflow (*n* = 2 donors). **d**, Overview of large-scale editing workflow. **e**, Expansion of cells after multiplexed editing. **f**, Number and composition of edited cells after expansion. **g**, Representative flow cytometry plots of TCR, CAR, B2M and CD47 expression. FITC, fluorescein isothiocyanate. Source data

To test whether SEED integration could be achieved at multiple loci simultaneously, we edited T cells with both *TRAC*-CAR and *B2M*-CD47 SEEDs (Fig. 2a–c). Cells were transduced at an MOI of 2 × 10^5^, an AAV dose chosen to mimic preclinical studies for CaMMouflage (NCT05722418), an ongoing clinical trial evaluating CRISPR-edited T cells with integrations at *TRAC* and *B2M* (ref. ^32^). High integration efficiencies were observed at both target loci with up to 55% of cells undergoing full editing (TCR^−^CAR^+^B2M^−^CD47^+^), confirming that multilocus integration could be attained with clinically relevant doses of AAV (Fig. 2b,c). Simultaneous immunomagnetic depletion with anti-TCR and anti-B2M antibodies further enriched for cells with transgene integrations, allowing for the isolation of a highly pure (89.4%) population of fully edited cells (Fig. 2b,c).

**Extended Data Fig. 6:**
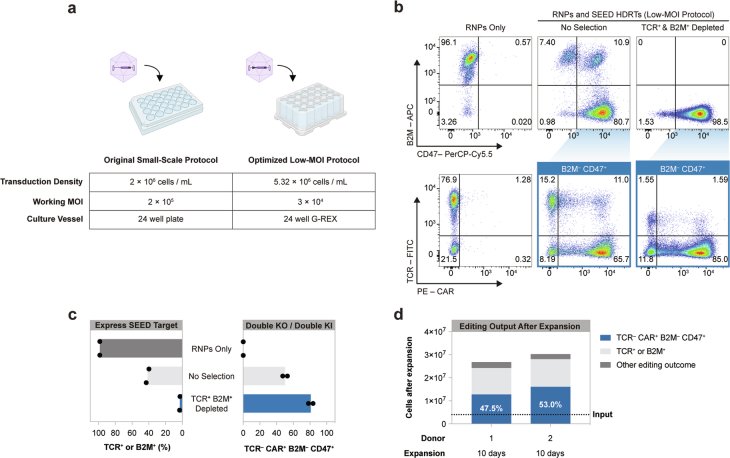
Efficient low-MOI editing can be achieved with high density transductions. **a**, Comparison of high and low MOI editing protocols. **b**–**d**, T cells were edited with RNPs targeting TRAC and B2M ( + M3814) and transduced with TRAC-CAR and B2M-CD47 SEED HDRTs in a G-REX plate at an MOI of 3 × 104 (per AAV). Edited cells were then immunomagnetically purified with anti-TCR and anti-B2M (n = 3 donors). **b**, Representative flow cytometry plots of TCR, CAR, B2M, and CD47 expression. **c**, Percentage of B2M+ or TCR+ cells and fully edited cells (TCR– CAR + B2M– CD47 + ). **d**, Number of cells generated from editing 4 × 106 cells followed by a 10-day expansion. Source data

To improve our ability to produce complex cell therapies at scale, we designed an optimized protocol using G-REX flasks on the basis of reports showing that AAV transduction is influenced by culture density (Extended Data Fig. 6a)^33^. Using this improved workflow, we were able to achieve complete editing at *TRAC* and *B2M* in ~50% of cells using 85% less virus (MOI of 3 × 10^4^) than our original protocol (Extended Data Fig. 6b,c). Robust expansion was observed following editing, enabling up to 1.6 × 10^7^ fully edited T cells to be generated from an initial input of 4 × 10^6^ cells after a 7-day expansion (Extended Data Fig. 6d). Multiplexed immunomagnetic depletion after expansion increased the fraction of fully edited cells from 50% to 81% with yields of up to 77% (Extended Data Fig. 4 and Extended Data Fig. 6b,c). These data demonstrate that SEED-Selection can be multiplexed to isolate homogenous cell products that have multiple desirable integrations.

### Multilocus low-MOI editing is efficient at clinical scale

To validate the scalability of our optimized editing protocol, we performed two clinical-scale manufacturing runs editing *TRAC* and *B2M* in 1.6 × 10^8^ primary human T cells using good manufacturing practice (GMP)-compatible reagents, equipment and processes (Fig. 2d). Complete editing was achieved in 71% of cells, with outputs of up to 2.5 × 10^9^ fully edited cells after only 7 days of expansion, indicating that multilocus integration strategies can be efficiently implemented in clinical settings (Fig. 2d–g).

### Epitope-edited human leukocyte antigen-independent TCRs are compatible with SEED-Selection

**Fig. 3:**
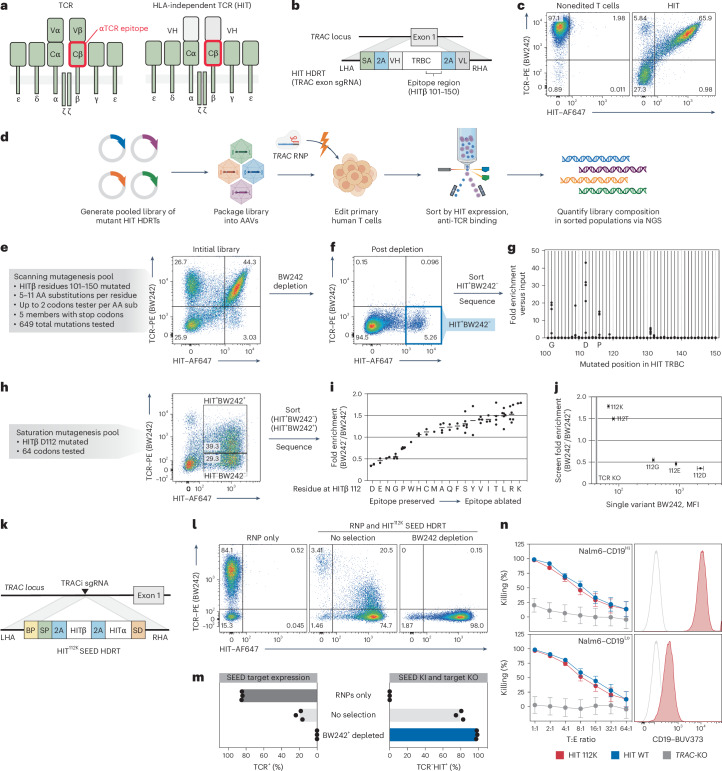
Epitope editing allows for TCR-based receptors to be used in TCR SEEDs. **a**, Diagram of a TCR and a HIT. **b**, Diagram of a *TRAC* exon-targeted HDRT encoding a HIT. **c**, Flow cytometry of anti-TCR (BW242) binding and HIT expression (anti-mouse F(ab′)_2_) in T cells with a HIT at *TRAC* (*n* = 1 donor). **d**, Screening workflow for epitope mapping. **e**–**g** T cells were edited to express a library of mutated HITs then purified with BW242. BW242 binding and HIT expression were quantified with flow cytometry before (**e**) and after (**f**) purification. HIT^+^ BW242^–^ cells were sorted (blue box) for sequencing (*n* = 1 donor). **g**, Enrichment of mutations in HIT^+^BW242^−^ cells (blue box in **f**) compared to the original library (**e**). Dots represent the average of tested codons for an amino acid. **h**, Flow cytometry of BW242 binding and HIT expression in T cells edited with a HIT β112 saturation mutagenesis pool. Boxes indicate sorted populations. **i**, Enrichment of mutations in HIT^+^BW242^−^ cells versus HIT^+^BW242^+^ cells. Dots represents enrichment for a single codon (*n* = 1 donor). Lines display the average enrichment for an amino acid. **j**, BW242 binding for HIT^+^ T cells edited with HIT variants (*n* = 3 donors) compared with average enrichment scores (from **i**). The black line indicates the BW242 median fluorescence intensity (MFI) for TCR^–^ cells. The MFI s.e.m. is represented by bars (HIT mutants) or the shaded gray area (TCR^–^). **k**, Diagram of a *TRAC* SEED HDRT encoding an epitope-edited HIT. **l**,**m**, T cells were edited with a HIT SEED (+M3814) and then purified with BW242 (*n* = 3 donors) **l**, Flow cytometry of BW242 binding and HIT expression **m**, Percentage of BW242^+^ and BW242^*−*^HIT^+^ cells. **n**, Cytotoxic activity of T cells with a nonmodified HIT (blue), epitope-edited HIT (red) or TCR knockout (gray) against Nalm6 lines (technical triplicate; data shown from one of four donors). Histograms show Nalm6 CD19 expression (flow cytometry). Unstained cells are shown in gray. Data are displayed as the mean ± s.e.m. T, target; E, effector. Source data

Synthetic immune receptors containing TRAC and TRBC domains, such as transgenic α/β TCRs^34^ or human leukocyte antigen (HLA)-independent TCRs (HITs)^35^, are highly sensitive to low antigen densities. However, these receptors are incompatible with TCR SEED-Selection as they are also bound by the anti-TCRα/β antibodies used to deplete nonmodified and partially edited cells (Fig. 3a–c). Structural analysis and high-throughput screening techniques have been used to design epitope-edited receptors that evade a specific antibody and remain functional^36–38^. We hypothesized that epitope editing of SEED payloads would allow for the enrichment of cells with a transgene that would otherwise be depleted in SEED-Selection.

Previous studies established that certain anti-TCRα/β antibody epitopes can be disrupted by murinizing a portion of the β constant domain (Cβ) of a TCR^24,39^. To identify single-amino-acid substitutions capable of disrupting antibody binding, we systematically substituted individual residues across a 50-aa span of the HIT Cβ in the form of a 649-member pooled knock-in library (Fig. 3d,e). After introducing the receptor pool into the *TRAC* locus of T cells, we immunomagnetically depleted cells that were bound by a GMP-grade anti-TCRα/β antibody (clone BW242/412; hereafter referred to as BW242) and flow-sorted for cells that retained high HIT expression (Fig. 3f). Library member abundance was then quantified by RNA-based next-generation sequencing (NGS) (Fig. 3g)^8^. While most library members were depleted, a subset of substitutions at Cβ residues G102, D112 or P116 were enriched, suggesting that these positions interact with BW242 and can be substituted to prevent HIT receptor depletion without compromising surface expression.

**Extended Data Fig. 7:**
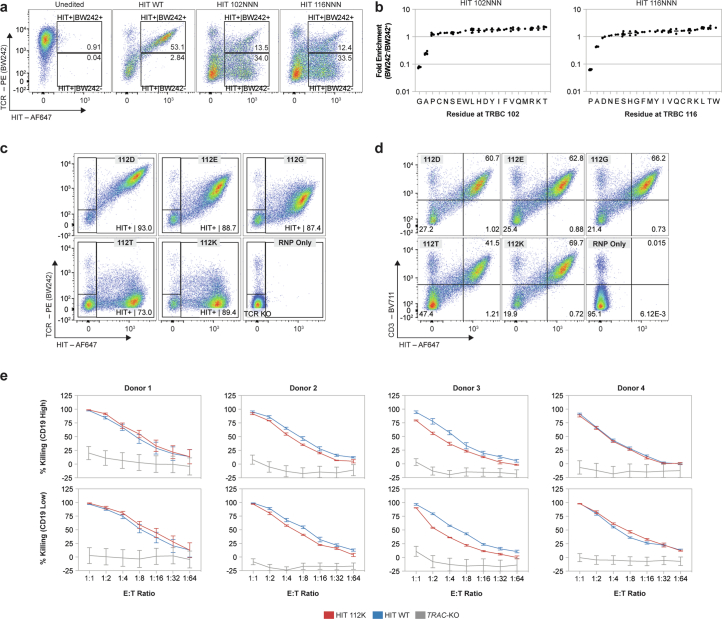
Characterization of individual and pooled libraries of epitope-edited HITs. **a**, Flow cytometry plots of BW242 binding and HIT expression (mouse F(ab’)2) in T cells edited with either a HIT β102 or β116 saturation mutagenesis pool. Boxes indicate sorted populations. **b**, Relative enrichment of mutations in HIT + BW242– cells versus HIT + BW242+ in cells from a. Each dot represents enrichment for a single codon. Bars represent the average enrichment of all codons for an amino acid (n = 1 donor). **c**, Representative flow cytometry plots of BW242 binding and HIT expression in T cells individually edited with TRAC-exon targeted HDRTs encoding HIT receptor variants. Median fluorescence intensity (MFI) values for BW242 binding (for Fig. 2j) were determined based on the HIT+ gated population (for transduced samples) or the TCR KO gated population (n = 3 donors). **d**, Representative flow cytometry plots of CD3 and HIT expression in T cells individually edited with TRAC-exon targeted HDRTs encoding HIT receptor variants (n = 3 donors), e, (continued from Fig. 3n) Cytotoxic activity of T cells with a non-modified HIT (blue), epitope-edited HIT (red), or TCR knockout (grey) against Nalm6 lines (technical triplicate, data shown from 1 of 4 donors). Data displayed as mean plus SEM. Source data

To catalog conservative and nonconservative substitutions at these key positions, we generated three pooled libraries of HIT receptors with saturation mutagenesis performed at Cβ residues 102, 112, or 116. Each library was individually introduced into T cells and HIT^+^ cells were flow-sorted on the basis of BW242 binding (Fig. 3h and Extended Data Fig. 7a). RNA-based NGS was then used to assess the relative enrichment of mutants in each bin as compared to the original library. Residues at each position were ranked from least to most conservative according to the ratio of enrichment between bins (Fig. 3i and Extended Data Fig. 7b). As expected, the native Cβ residue (G102, D112 and P116) at each position was the most conservative. Almost all substitutions at 102 and 116 were preferentially enriched in the BW242^−^ bin, suggesting that the native residues at these positions are required for optimal BW242 binding (Extended Data Fig. 7b). In total, 14 of 19 substitutions at Cβ residue 112 were also enriched in the BW242^–^ bin (Fig. 3i). However, substitutions to E, N, G, P and W were preferentially enriched in the BW242^+^ bin. The most conservative substitution at Cβ residue 112 preserved the charge (D to E) whereas the least conservative ones switched the charge (D to K or D to R), suggesting that electrostatic interactions with D112 contribute to BW242 binding.

To confirm that this sequencing-based approach for epitope mapping predicts changes in antibody binding, we generated five HIT receptors with different amino acids at Cβ residue 112 and individually introduced them into T cells. BW242 binding was assessed by flow cytometry and compared to the enrichment ratios obtained from the Cβ residue 112 saturation mutagenesis screen (Fig. 3j and Extended Data Fig. 7c). Sequencing-based epitope mapping correctly ranked the panel of substitutions on the basis of epitope conservation and accurately detected variation in BW242 binding across the dynamic range. HIT and CD3 surface expression was maintained across receptor designs, confirming that variations in BW242 binding were not because of alterations in HIT expression or assembly with CD3 chains (Extended Data Fig. 7d). Of the five tested mutants, HIT K112 had the least BW242 binding and was selected for functional characterization (Fig. 3j).

We introduced a CD19-specific HIT^K112^ into a *TRAC*-targeted SEED and immunomagnetically purified edited T cells. Depletion with BW242 resulted in complete removal of cells with endogenous TCRs and allowed for the recovery of a pure (>98%) population of HIT^+^ cells with yields of up to 68% (Fig. 3k–m and Extended Data Fig. 4). As the HIT receptor was developed to have the capacity to target tumor cells expressing low antigen densities^35^, we assessed receptor function through cytotoxicity assays with target cell lines expressing high or low levels of CD19. HIT^K112^ performed similarly to nonmodified HIT (HIT^WT^) against lines with high and low antigen density, confirming that epitope editing allows HIT^+^ cells to be enriched through SEED-Selection without compromising receptor function (Fig. 3n and Extended Data Fig. 7e).

### Epitope-edited TCRs enable minimization of mispairing

T cells can be engineered to target a specified peptide-MHC through the expression of a transgenic TCR^34^. However, mispairing between endogenous and transgenic TCR chains can occur when a transgenic TCR is expressed in an otherwise nonedited T cell^24,40^. Because all TCR chains compete for the same pool of CD3 molecules during assembly, mispairing can reduce the surface expression of the correctly paired transgenic TCR^24,40^. Mispairing-associated autoreactivity has also been reported in mouse models but the clinical importance of this phenomenon has not yet been established^40^.

**Fig. 4:**
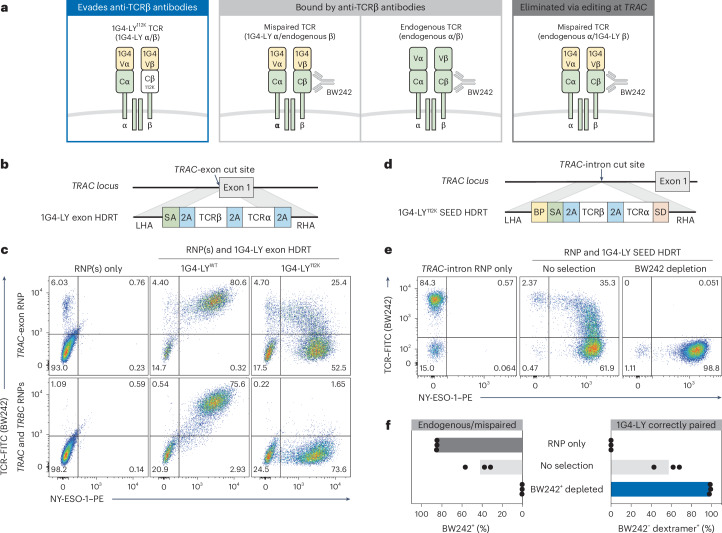
SEED-Selection depletes *TRAC*-edited TCR-swapped cells that express mispaired TCRs. **a**, Schematic of possible TCR pairs in nonedited and *TRAC*-edited T cells engineered to express the 1G4-LY^K112^ TCR (specific for NY-ESO-1). **b**, Diagram of a *TRAC* exon-targeted HDRT encoding 1G4-LY. **c**, Flow cytometry plots of BW242 binding and NY-ESO-1 dextramer binding in CD8^+^ cells. Editing was performed with RNPs targeting *TRAC* or *TRAC/TRBC* and HDRTs (**a**) encoding nonmodified (1G4-LY^WT^) or epitope-edited (1G4-LY^K112^) versions of 1G4-LY (*n* = 2 donors). **d**, Diagram of a *TRAC* intron-targeted SEED HDRT encoding 1G4-LY. **e**,**f**, T cells were edited with a 1G4-LY SEED (+M3814) (**d**) and immunomagnetically purified with BW242 (*n* = 3 donors). **e**, Flow cytometry plots of BW242 binding and NY-ESO-1 dextramer binding for cells gated on CD8^+^. **f**, Percentage of cells with an endogenous or mispaired TCRs (BW242^+^) or correctly paired 1G4-LY (dextramer^+^BW242^*–*^). Source data

Mispairing is a nonrandom process influenced by properties of the transgenic TCR and each T cell’s endogenous TCR and some combinations of TCR chains pair inefficiently^41^. In *TRAC*-edited cells engineered to express a transgenic TCR, mispairing occurs between the transgenic TCRα and endogenous TCRβ (ref. ^24^). Therefore, we hypothesized that editing the BW242 epitope on a transgenic TCRβ chain could allow cells that express a correctly paired transgenic TCR (which should evade BW242 binding) to be distinguished from cells that express a mispaired TCR (which should retain BW242 binding) (Fig. 4a).

To test this hypothesis, we generated an epitope-edited variant of 1G4-LY: a clinically validated, affinity-matured, transgenic TCR that targets NY-ESO-1 (SLLMWITQC) on HLA-A*02 (ref. ^42^). We designed *TRAC* exon-targeted HDRTs to simultaneously disrupt the endogenous TCRα and facilitate expression of a nonmodified 1G4-LY (1G4-LY^WT^) or an epitope-edited variant (1G4-LY^K112^) (Fig. 4b). Each HDRT was introduced into CD8^+^ T cells edited with an RNP targeting *TRAC*. Multiplexed editing of *TRAC* and *TRBC* (TCRβ constant) was also performed to produce T cells where 1G4-LY was expressed and both endogenous TCR chains were disrupted, ensuring exclusive expression of correctly paired 1G4-LY.

**Extended Data Fig. 8:**
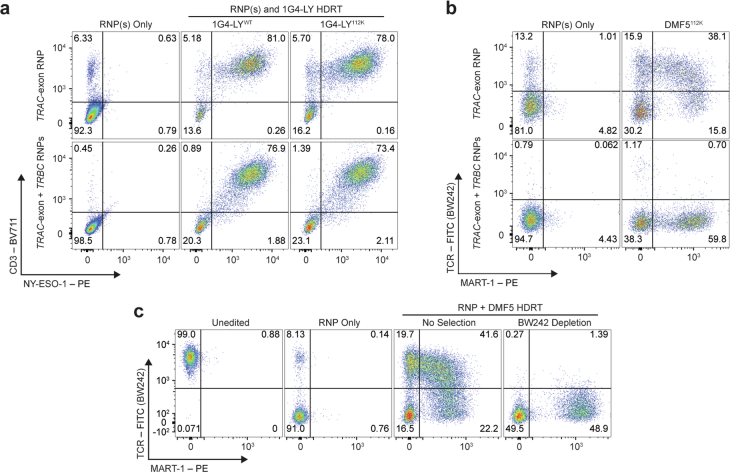
Validation of epitope editing in transgenic TCRs. **a**, Flow cytometry plots quantifying CD3 expression and NY-ESO-1 dextramer binding in CD8+ cells. Editing was performed with RNPs targeting TRAC or TRAC/TRBC and HDRTs encoding non-modified (1G4-LYWT) or epitope-edited (1G4-LY112K) versions of 1G4-LY (n = 2 donors). **b**, Flow cytometry plots quantifying BW242 binding and MART-1 dextramer binding in CD8+ cells. Editing was performed with RNPs targeting TRAC or TRAC/TRBC and an HDRT encoding an epitope-edited version of a MART-1 TCR (DMF5) (n = 1 donor). **c**, Flow cytometry plots of BW242 binding and MART-1 dextramer binding in CD8 + –gated T cells edited with a MART-1 TCR HDRT after immunomagnetic BW242 depletion (n = 1 donor).

NY-ESO-1 dextramer binding correlated with BW242 binding in cells expressing 1G4-LY^WT^, as expected (Fig. 4c). In contrast, *TRAC*-edited cells engineered to express 1G4-LY^K112^ exhibited a distinct population with correct TCR pairing (dextramer^+^BW242^−^) and a separate population with apparent TCR mispairing (dextramer^+^BW242^+^). In agreement, this population was eliminated by concurrent knockout of the endogenous TCRβ chain (Fig. 4c). Dextramer^+^ cells in all conditions expressed similar levels of CD3, confirming that variations in BW242 binding were not caused by differences in TCR assembly (Extended Data Fig. 8a). We also used epitope editing to identify and deplete mispaired *TRAC*-edited cells engineered to express a transgenic TCR (DMF5) targeting MART1 (melanoma antigen recognized by T cells 1), supporting the generalizability of this approach (Extended Data Fig. 8b,c)^43^.

To test whether an epitope-edited TCR could be enriched through SEED-Selection, we introduced 1G4-LY^K112^ into a *TRAC*-targeted SEED and edited CD3^+^ T cells (Fig. 4d). Depletion with BW242 resulted in efficient removal of cells with endogenous or mispaired TCRs and the recovery of a highly pure (>98%) population of correctly paired 1G4-LY^+^ cells with yields of up to 60% (Fig. 4e,f and Extended Data Fig. 4). This result suggests that SEED-Selection could be used to deplete T cells with undesired specificities without the need to perform simultaneous editing at *TRAC* and *TRBC*.

### SEED enables simultaneous coreceptor and TCR swapping

**Extended Data Fig. 9:**
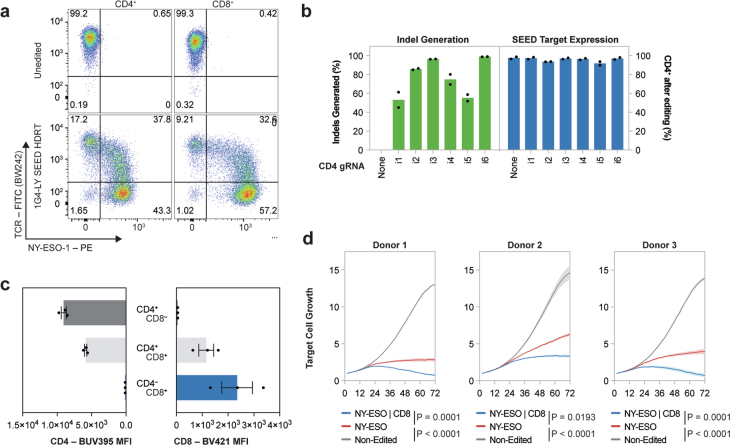
SEED-Selection facilitates co-receptor swapping. **a**, BW242 binding and NY-ESO-1 dextramer binding in CD4+ and CD8+ cells edited with a 1G4-LY112K SEED. **b**, Assessment of CD4 expression by flow cytometry and indel generation by bulk genomic DNA sequencing in T cells after editing with intron-targeted gRNAs (n = 2 donors). **c**, Expression of CD4 or CD8 in subpopulations of non-purified CD8 SEED edited cells. MFI: median fluorescence intensity. Data displayed as mean plus SEM (n = 3 donors). **d**, Assessment of cytotoxic activity of CD4 + T cells against NY-ESO-1 + A375 target cells (continued from Fig. 5h), Non-edited T cells (dark gray), 1G4-LY SEED edited (red), 1G4-LY SEED and CD8 SEED edited (blue). Data displayed as mean (dark line) plus SEM (shaded area) (technical triplicate, n = 3). Significance was assessed at 72 hours using a repeated-measures one-way ANOVA and Turkey’s multiple comparison test. Source data

Recent studies have emphasized the role of CD4^+^ T cells as contributors to long-lasting immune responses to tumors^44^. However, the MHC-I-restricted TCRs commonly isolated for transgenic TCR therapies perform suboptimally in the absence of the CD8α/β coreceptor^45–47^. Although 1G4-LY has been demonstrated to undergo coreceptor-independent activation^42^, we observed that dextramer binding was markedly reduced in edited CD4^+^ T cells as compared to edited CD8^+^ T cells (Extended Data Fig. 9a). Therefore, we sought to develop a strategy for isolating CD4^+^ T cells edited to express both CD8α/β and 1G4-LY.

**Fig. 5:**
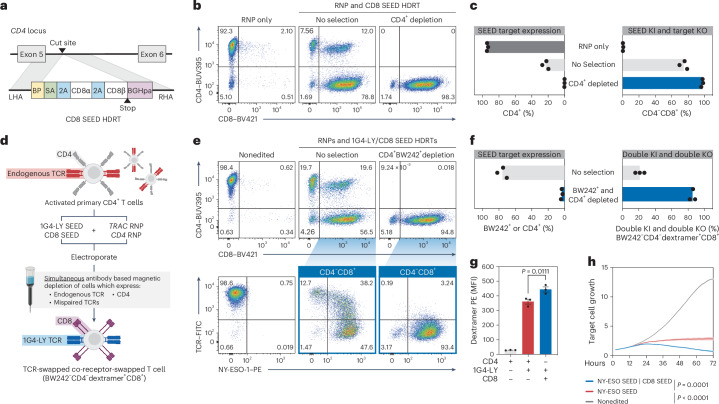
SEED-Selection enables the enrichment of TCR-swapped, coreceptor-swapped cells. **a**, Diagram of a *CD4* intron-targeted SEED HDRT encoding CD8α/β. **b**,**c**, CD4^+^ T cells were edited with a CD8 SEED (**a**) (+M3814) and immunomagnetically purified with anti-CD4 (*n* = 3 donors). **b**, Flow cytometry plots of CD4 and CD8 expression. **c**, Percentage of CD4^+^ and CD4^−^CD8^+^ cells. **d**–**h** CD4^+^ T cells were edited with 1G4-LY and CD8 SEEDs (+M3814) and immunomagnetically purified with BW242 and anti-CD4 (*n* = 3 donors). **d**, Overview of editing and selection strategy. **e**, Flow cytometry plots of CD4 and CD8 expression and BW242 and NY-ESO-1 dextramer binding. **f**, Percentage of cells that express CD4 or an endogenous or mispaired TCR (BW242^+^). Percentage of fully edited cells with minimal mispairing (BW242^−^CD4^−^dextramer^+^CD8^+^). **g**, Assessment of NY-ESO-1 dextramer binding by flow cytometry in CD4^+^CD8^−^BW242^−^1G4-LY^+^ cells (red), CD4^−^CD8^+^BW242^−^1G4-LY^+^ cells (blue) and nonedited CD4^+^ T cells (gray) (*n* = 3 donors). Data are displayed as the mean ± s.e.m. **h**, Assessment of cytotoxic activity of CD4^+^ T cells against NY-ESO-1^+^ A375 target cells (technical triplicate; data shown from one of four donors). Nonedited T cells, dark gray; 1G4-LY SEED-edited, red; 1G4-LY SEED-edited; CD8 SEED-edited, blue. Data are displayed as the mean (bold line) ± s.e.m. (shaded area). Significance was assessed at 72 h. The significance in **g**,**h** was assessed using a repeated-measures one-way ANOVA and Turkey’s multiple-comparisons test. Source data

As CD4 should be superfluous in cells engineered to express an MHC-I-restricted TCR, we screened for nondisruptive gRNAs targeting the *CD4* locus and designed a SEED HDRT to co-deliver CD8α and CD8β (Fig. 5a and Extended Data Fig. 9b). Integration-mediated CD4 disruption (CD4^−^CD8^+^) was achieved in >75% of HDRT-transduced CD4^+^ cells (Fig. 5b,c). Full disruption of CD4 requires both alleles to be nonfunctional. Correspondingly, a population that coexpressed intermediate levels of CD4 and CD8 was observed after editing, consistent with monoallelic SEED integration (Extended Data Fig. 9c). Immunomagnetic CD4 depletion allowed for the recovery of a pure (>98%) population of cells with biallelic SEED integration (CD4^−^CD8^+^) with yields of up to 79% (Fig. 5b,c and Extended Data Fig. 4).

To assess whether the performance of 1G4-LY in CD4^+^ cells was improved through overexpression of CD8α/β, we edited T cells with a *TRAC*-targeted 1G4-LY^K112^ SEED alone or with a *CD4*-targeted CD8α/β SEED (Fig. 5d). Each cell population was immunomagnetically purified with anti-TCR (BW242) and anti-CD4 antibodies to enrich for transgene integration and to deplete cells with mispaired and endogenous TCRs (Fig. 5d–f and Extended Data Fig. 4). As expected, cells that expressed both 1G4-LY^K112^ and CD8α/β exhibited increased NY-ESO-1 dextramer binding in comparison to cells that expressed 1G4-LY^K112^ alone (Fig. 5g). Furthermore, in a longitudinal cytotoxicity assay, coexpression of CD8α/β improved the control of A375, a melanoma cell line that endogenously expresses NY-ESO-1 (Fig. 5h and Extended Data Fig. 9d).

**Fig. 6:**
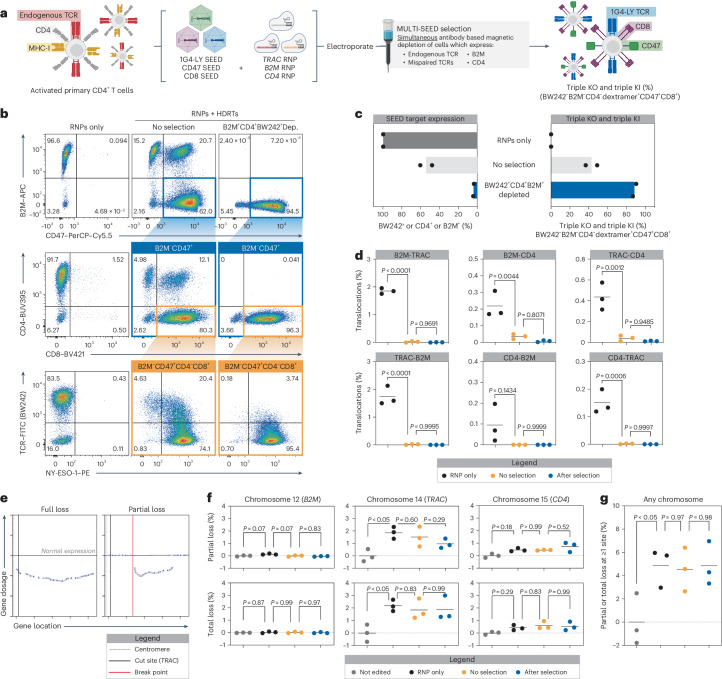
SEED-Selection enables the isolation of complex cell therapies without enriching for translocations or chromosomal loss between target loci. **a**–**c**, CD4^+^ T cells were edited with RNPs targeting *TRAC*, *B2M* and *CD4* (+M3814) and transduced with SEEDs encoding 1G4-LY, CD8 and CD47, respectively. Edited cells were then immunomagnetically purified with BW242, anti-CD4 and anti-B2M *(n* = 2 donors*)*. **a**, Diagram of the workflow for multiplexed editing and enrichment. **b**, Flow cytometry plots of SEED target expression (endogenous TCR, CD4 and B2M), SEED payload expression (CD8 and CD47) and NY-ESO-1 dextramer binding. **c**, Percentage of cells expressing any SEED target, a mispaired TCR (BW242^+^, CD4^+^ or B2M^+^) or triple-knockout or triple-knock-in cells with correct 1G4-LY pairing (BW242^−^B2M^−^CD4^−^dextramer^+^CD47^+^CD8^+^). **d**, Assessment of balanced translocations among *TRAC*, *B2M* and *CD4* after multiplexed editing (+M3814) and SEED-Selection by ddPCR after a 9-day expansion (*n* = 3 donors). **e**–**g**, partial and total chromosome loss was assessed in after multiplexed editing and SEED-Selection by single-cell RNA sequencing (*n* = 3 donors). **e**, Representative gene expression profiles of cells with partial or complete loss of chromosome 14 after multiplexed editing. **f**, Normalized frequency of cells with partial and total chromosomal loss. **g**, Normalized frequency of cells with one or more chromosomal abnormalities. The significance in **d**,**f**,**g** was assessed using a repeated-measures one-way ANOVA and Turkey’s multiple-comparisons test. Source data

**Extended Data Fig. 10:**
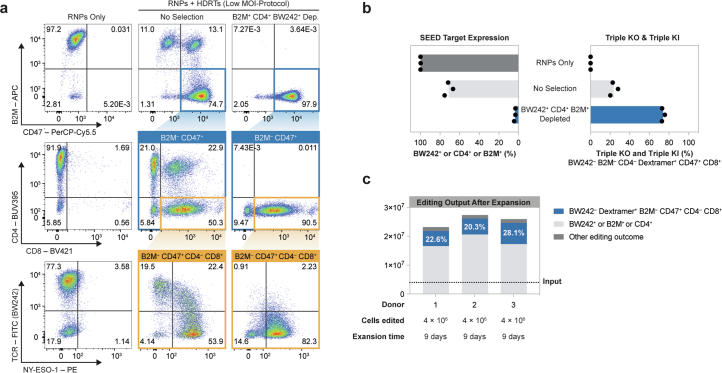
Optimized multi-locus low-MOI editing at TRAC, B2M, and CD4. CD4 + T cells were edited with RNPs targeting TRAC, B2M, and CD4 ( + M3814) and transduced with SEEDs encoding 1G4-LY, CD8, and CD47 in a G-REX plate at an MOI of 3 × 104 (per AAV). Edited cells were then immunomagnetically purified with BW242, anti-CD4, and anti-B2M (n = 3 donors). **a**, Flow cytometry plots of SEED target expression (endogenous TCR, CD4, B2M), SEED payload expression (CD8, CD47), and NY-ESO-1 dextramer binding. **b**, Percentage of cells expressing any SEED target or a mispaired TCR (BW242+ or CD4+ or B2M + ) or triple knockout/triple knock-in cells with correct 1G4-LY pairing (BW242– B2M– CD4– Dextramer+ CD47 + CD8 + ). **c**, Number of cells generated from editing 4 × 106 cells followed by a 9-day expansion. Source data

To validate the ability of SEED-Selection to isolate fully edited cells after complex editing, we sought to simultaneously select for cells with clinically desirable transgenes integrated at three loci (Fig. 6a). CD4^+^ cells were edited with RNPs targeting *TRAC*, *B2M* and *CD4* and transduced with SEEDs encoding 1G4-LY^K112^, CD47 and CD8. After editing, cells expressing any combination of endogenous TCR, mispaired TCR, B2M and CD4 were immunomagnetically removed in a single step (Fig. 6b,c). Depletion of all target markers was efficient, resulting in the recovery of highly pure (up to 90%) populations of fully edited BW242^−^B2M^−^CD4^−^1G4-LY^+^CD47^+^CD8^+^ cells with yields of up to 50% (Fig. 6b,c and Extended Data Fig. 4). Multiplexed SEED-Selection was also performed on cells transduced with our low-MOI protocol, where immunomagnetic selection increased the percentage of fully edited nonmispaired cells from 24% to 70% (Extended Data Fig. 4 and Extended Data Fig. 10a–c).

### Evaluation of genome stability after multilocus SEED editing

Rearrangements such as translocations and chromosome loss have been observed in gene-edited primary cells^48–50^. To understand how SEED-Selection influences translocation frequency, we designed an array of digital droplet PCR (ddPCR) assays and quantified balanced translocations 9 days after simultaneous editing at *TRAC*, *B2M* and *CD4*. When editing was performed in the absence of HDRTs, individual translocation frequencies ranged from 0.1% to 1.9% (Fig. 6d). As previously observed, translocation frequencies decreased significantly when editing was performed with HDRTs (Fig. 6d)^51^. Translocations could hypothetically result in integration-independent disruption of SEED target expression. However, further isolation of fully edited cells by SEED-Selection did not significantly alter translocation frequencies, suggesting that cells with translocations may often retain expression of at least one SEED target (Fig. 6d). These data build on previous findings that unintended editing outcomes can be prevented through the addition of an HDRT and validate that SEED-Selection does not enrich for balanced translocations.

To determine whether SEED-Selection enriches for genome rearrangements that result in integration-independent SEED target disruption through partial or complete chromosome loss, we used a previously described single-cell RNA sequencing-based workflow to evaluate the frequency of these events at chromosome 12 (*B2M)*, chromosome 14 (*TRAC*) and chromosome 15 (*CD4*) in comparison to a nonedited control (Fig. 6e). Both partial and complete chromosome loss in HDRT edited cells was more frequent at chromosome 14 (1.5% partial and 1.8% total) versus chromosome 12 (<0.01% partial and 0.02% total) or chromosome 15 (0.5% partial and 0.6% total), which might be because of the proximity of the *TRAC* locus to the centromere, correlating with the increased frequency of integration-independent SEED target disruption observed with *TRAC* gRNAs (Fig. 6e,f). The occurrence of partial and complete loss of these chromosomes was not significantly affected by the presence of an HDRT during editing or by SEED-Selection (Fig. 6f). To globally evaluate changes in genome integrity, we also quantified the frequency of cells with at least one partial or total deletion involving any autosomal chromosome and found no significant differences associated with SEED-Selection (Fig. 6g). These results further validate that large-scale genomic rearrangements involving target loci are not enriched through SEED-Selection.

## Discussion

SEED-Selection has many characteristics that are suited to clinical applications. SEED-Selection does not require the expression of exogenous proteins such as drug resistance cassettes, which can provoke an immune response^52^. The reductive nature of SEED-Selection also leaves isolated cells unlabeled and allows for multiple SEEDs to be simultaneously enriched. Furthermore, SEED HDRTs minimize the need to introduce DSBs at irrelevant sites in applications where the disruption of one or more surface proteins is desired, such as for allogeneic cell therapy manufacturing.

SEED-Selection is highly effective at enriching cells with intended editing outcomes, enabling therapeutic doses of fully edited cells to be delivered with reduced contamination from unwanted cell populations. Additionally, as SEED-Selection is based on immunomagnetic negative selection, it is unlikely that the purification process directly alters the viability and genome integrity of the product. While we were able to isolate populations with biallelic transgene integration in >98% of cells after editing at a single locus, cells with unintended editing outcomes may evade depletion through integration-independent SEED target disruption. We demonstrate that SEED-Selection does not enrich for balanced translocations or chromosome loss when editing is performed at *TRAC*, *B2M* and *CD4*. However, additional validation should be performed for strategies targeting other loci.

Several considerations apply to the design and application of SEED HDRTs and SEED-Selection. SEED HDRTs must be integrated at loci that encode cell surface proteins that are not required for cell survival. Additionally, the designs we tested use endogenous promoters that maximize HDRT payload capacity but may not provide optimal transcriptional regulation for some payloads. In applications where endogenous regulation is not suitable, an alternative SEED cassette could be used where a stop codon is introduced after the SA and transgene expression is driven by a user-selected exogenous promoter.

During SEED-Selection, potentially functional cells with monoallelic integrations are depleted. However, we demonstrate that editing conditions can be optimized so that biallelic integration is the primary editing outcome and describe an optimized GMP-compatible editing workflow for achieving high-efficiency biallelic integration during multiplexed editing. Fully edited cells can also be lost during the purification process during washes or because of nonspecific binding. Therefore, SEED-Selection is most practical in applications where immunomagnetic purification is already required or where biallelic transgene integration is essential for therapeutic activity. Alternatively, SEED-Selection could be used to efficiently screen for complex editing outcomes in stem cells or other immortalized populations.

Our testing of SEED-Selection was limited to research-scale purifications (<2 × 10^7^ cells per purification) but protocols for large-scale multitarget immunomagnetic depletion with SEED-compatible reagents have been developed for the CliniMACS Prodigy, an automated cell processing platform that is commonly used in clinical manufacturing^53,54^. While we demonstrate that up to three SEEDs can be enriched in a single round of selection, immunomagnetic panels targeting ten or more surface proteins are routinely used in laboratory and clinical settings^55^. Therefore, we envision that more complex SEED-Selection strategies could be implemented as genome-editing technologies advance. We expect that this approach will streamline manufacturing for current products and enable the development of more advanced cellular therapies.

## Methods

### T cell isolation and culture

Primary adult blood cells from anonymous healthy human donors were purchased as leukapheresis packs (StemCell Technologies) and cryopreserved. Specific lymphocytes were isolated from thawed aliquots using EasySep isolation kits for CD3^+^, CD4^+^ or CD8^+^ T cells (StemCell Technologies). Isolated T cells were cultured at an initial density of 10^6^ cells per ml in X-VIVO 15 medium (Lonza) supplemented with human serum (5%, Gemini), penicillin–streptomycin (1%, Gibco), interleukin (IL-7; 5 ng ml^−1^, Miltenyi) and IL-15 (5 ng ml^−1^, Miltenyi). After isolation, cells were stimulated for 2 days with anti-human CD3/CD28 magnetic Dynabeads (Thermo Fisher Scientific) using a 1:1 bead-to-cell ratio.

### NK cell isolation and culture

Frozen primary human NK cells (StemCell Technologies) were thawed and activated overnight in RPMI (Gibco) supplemented with FBS (10%), nonessential amino acids (1%, Gibco) and IL-2 (1,000 U per mL, Peprotech), as previously described, before use in functional assays^28^.

### HDRT design

Sequences for individually tested HDRTs and site saturation mutagenesis pools are provided in Supplementary Table 2. Additional annotations for SEED HDRT designs are provided in Supplementary File 1. Splicing elements at the 5′ end of SEEDs (which include the polypyrimidine tract, branchpoint (BP) sequence and SA) were derived from the chimeric intron included in the pCI mammalian expression vector (Promega). We used a P2A sequence to prematurely truncate the SEED target and facilitate expression of a transgene payload. We selected integration sites between the signal peptide and transmembrane domain of the SEED target, so that SEED target surface expression would be disrupted upon HDRT integration. In most SEED designs, the 3′ end of the SEED included an additional P2A followed by the SD sequence of the preceding exon. This allows the transgene to be expressed with the endogenous poly(A) sequence of the SEED target and conserves HDRT cargo capacity. In SEED HDRTs encoding transgenic TCRs or HITs, the 3′ P2A was excluded to allow the HIT/TCRα chain to be completed using the endogenous *TRAC* sequence. Alternatively, our *CD4*-targeted SEED did not include a final P2A or SD sequence and instead relied on a bovine growth hormone poly(A) (BGHpa) signal. Where necessary, additional nucleotides were added downstream of the SA and/or upstream of the SD to maintain the reading frame of the spliced transcript.

### AAV production

AAV plasmids were packaged into AAV6 by transfection of HEK293T cells (American Type Culture Collection) and purified using iodixanol gradient ultracentrifugation. Titers were determined by qPCR on AAV samples treated with DNase I (New England Biolabs (NEB)) and digested with proteinase K (Qiagen). HDRTs targeting *TRAC* exon 1 were quantified using primers targeting the left homology arm of the HDRT, while all other AAVs were quantified using primers targeting the inverted terminal repeat sequences (Supplementary Table 3). qPCR was performed with SsoFast EvaGreen supermix (BioRad) on a StepOnePlus real-time PCR system (Applied Biosystems).

### RNP formulation

gRNA sequences are provided in Supplementary Table 4. For most experiments, RNP was generated by incubating single gRNAs (sgRNAs; Synthego) with Cas9 protein (40 µM, Berkeley QB3 MacroLab) at a 2:1 (sgRNA:Cas9) molar ratio for 15 min at 37 °C. For intron gRNA screening experiments, RNPs were produced by complexing a two-component gRNA (Edit-R, Dharmacon Horizon) to Cas9 protein with the addition of a poly(l-glutamic acid) (Sigma) electroporation enhancer, as previously described^11^. When multiple loci were targeted, RNPs were individually complexed and then mixed shortly before electroporation.

### T cell editing

For each electroporation, 2 × 10^6^ cells were resuspended in P3 buffer (Lonza), mixed with RNPs and added to a 96-well nucleofection plate (Lonza). RNP amounts per electroporation varied as a function of the number of loci targeted: one locus, 60 pmol of RNP; two loci, 60 pmol of each RNP (120 pmol in total); three loci, 53 pmol of each RNP (159 pmol in total). P3 buffer volume was adjusted so that the total volume of each reaction was 23 μl.

Cells were electroporated using a Lonza 4D-Nucleofector 96-well unit (EH-115). Prewarmed X-VIVO 15 medium (without human serum) was then added to achieve a density of 2 × 10^6^ live cells per ml, assuming a one-third loss of viability after electroporation. AAV6 encoding HDRTs was added to cultures shortly after editing. For most experiments, an MOI of 2 × 10^5^ was used. After an overnight incubation, edited cells were resuspended in fresh complete medium. Edited cells were subsequently expanded, keeping a density of 10^6^ cells per ml.

### Optimized G-REX transduction protocol

For small-scale G-REX experiments, 4 × 10^6^ T cells were electroporated as described above. Prewarmed X-VIVO 15 medium (with M3814 and without human serum) was then added to achieve a density of 5.28 × 10^6^ live cells per ml, assuming a one-third loss of viability after electroporation. Edited cells were then transferred to a single well of 24-well G-REX plate (Wilson Wolf) and transduced with AAVs at an MOI of 3 × 10^4^ per construct. After an overnight incubation, edited cells were resuspended in 8 ml of fresh complete medium. Then, 3 days after editing, 6 ml of medium was removed from each well (without disturbing the cells) and replaced with 6 ml of fresh complete medium. Cells were then expanded for an additional 6–7 days.

### GMP editing workflow

For GMP-compatible, clinical-scale experiments, primary T cells from healthy donors were isolated and activated with CTS Dynabeads CD3/CD28 using a 3:1 bead-to-target cell ratio on the Gibco CTS DynaCellect magnetic separation system (Thermo Fisher Scientific). The isolated T cells were cultured for 2 days in X-VIVO 15 medium (Lonza) supplemented with human serum (5%, Grifols), IL-7 (20 U per ml, Miltenyi) and IL-15 (100 U per ml, Miltenyi).

Dry aliquots of sgRNAs (Synthego) were resuspended with duplex buffer to a concentration of 320 μM and incubated for 15 min at 37 °C. The sgRNAs were then incubated with Cas9 protein (62.5 µM, Aldevron) at a 4:1 (sgRNA:Cas9) molar ratio for 15 min at 37 °C. RNPs were individually complexed and then mixed shortly before electroporation.

For each electroporation, 8 × 10^7^ cells were resuspended in electroporation buffer (MaxCyte), mixed with 1,200 pmol of each RNP and added to an R-1000 processing assembly (MaxCyte) at a volume of 600 μl. Cells were electroporated using the MaxCyte GTx electroporation system (Expanded T Cell 4-2). Then, 400 μl of prewarmed medium (without human serum) was immediately added and the cells were incubated at 37 °C for 30 min. The cells were plated in a G-Rex 100M open system (Wilson Wolf) at a density of 5.32 × 10^6^ live cells per ml (assuming a one-third loss of viability after electroporation) with AAV6 encoding HDRTs in complete medium (without human serum) supplemented with M3814 (1 μM, ChemieTek). After overnight incubation, edited cells were resuspended in 1 L of fresh complete medium to expand.

### Flow cytometry and sorting

Flow cytometry was performed on a BD FACSymphony Fortessa X-50 or an Attune NxT. Cell sorting was performed on a BD FACSAria. Cells were resuspended in fluorescence-activated cell sorting buffer (PBS, 2% FBS and 1 mM EDTA) and stained with antibodies or dextramer (Supplementary Table 5). Antibodies were diluted as follows: 1:25 (B6H12–PerCP-Cy5.5), 1:50 (NY-ESO-1 dextramer and MART1 dextramer) and 1:100 Zombie violet (BioLegend) or Ghost Dye red (Tonbo) were used in experiments where viability was assessed by flow cytometry. In experiments where HIT or CAR expression was assessed, cells were initially stained with anti-mouse F(ab′)_2_ and then blocked with mouse serum (MilliporeSigma) before antibody staining was performed. In experiments where anti-B2M and MHC-I dextramers were both used, cells were stained with antibodies first, washed and then stained with dextramer. Flow cytometry analysis was performed in FlowJo (BD Biosciences). Representative gating strategies are provided in the Supplementary Fig. 1–6.

### Intronic gRNA screening

Activated human T cells were edited with individual gRNAs and cultured for 3 days. Surface marker expression was then assessed by flow cytometry and genomic DNA was isolated using QuickExtract (Epicenter). PCR amplification of cut site regions was performed with KAPA HiFi polymerase (Kapa Biosystems) according to the manufacturer-provided protocol. Amplicons were purified using solid-phase reversible immobilization (SPRI) beads (Beckman) and Sanger-sequenced (Quintara Biosciences). The resulting sequencing files were aligned for detection of indels using the ICE Analysis webtool (Synthego, https://ice.synthego.com/#/).

### SEED engineering and selection

For experiments with SEED HDRTs, M3814 (1 μM, ChemieTek) was added to the recovery medium after editing, unless otherwise specified. All purifications were performed 7–10 days after editing. Before selection, cell density and viability were assessed using a Countess II automated cell counter (Thermo Fisher Scientific). Cells were then centrifuged, resuspended in MACS buffer (80 μl per 10^7^ cells; PBS, 0.5 M EDTA and 2% BSA) and incubated with a biotin-conjugated antibody (Supplementary Table 5) targeting the marker of interest (20 μl per 10^7^ cells) for 10 min at 4 °C. In experiments where multiple markers were simultaneously depleted, cells were incubated with a master mix of antibodies (20 μl of each antibody per 10^7^ cells) and MACS buffer volume was adjusted to maintain a consistent incubation volume of (100 μl per 10^7^ cells). Cells were then washed, resuspended in MACS buffer (80 μl per 10^7^ cells) and incubated with anti-biotin microbeads (20 μl per 10^7^ cells, Miltenyi) for 15 min at 4 °C. Labeled cells were loaded onto Miltenyi MACS columns and processed according to the manufacturer-provided protocol. Cell density in the flowthrough from the column was assessed and isolated cells were centrifuged and resuspended in complete T cell medium for culture. The SEED target and transgene payload expression was evaluated within 24 h using flow cytometry. Cell counts used to estimate purification yield are included in Supplementary Table 1.

### *B2M* integration site genomic DNA PCR

T cells were edited with *B2M* intron-targeted RNP (i4) and SEED HDRT encoding CD47. Edited cells were then expanded for 7 days and immunomagnetically purified with anti-B2M antibody. Genomic DNA was isolated from nonedited cells, nonpurified edited cells and purified edited cells using a NucleoSpin Tissue Kit (Macherey-Nagel). The HDRT integration site was PCR-amplified from genomic DNA using Q5 high-fidelity polymerase (NEB), with an expected amplicon size of ~1 kb for nonedited *B2M* alleles and ~2 kb for alleles with HDRT integration. Primers were designed to target sequences upstream and downstream of the HDRT homology arms to avoid amplification of the nonintegrated repair template. Amplicon size was assessed using gel electrophoresis (0.8% agarose, 125 V for 50 min) with SYBR Safe DNA stain (Thermo Fisher Scientific) using the 1-kb Plus DNA ladder (NEB). Gel imaging was performed on a FluorChem M System (Cell Biosciences). Unprocessed gel images and a diagram of the primer binding sites are provided in Supplementary Fig. 7 and the Source Data.

### HIT scanning mutagenesis library design and synthesis

A pooled library of oligos encoding Cβ residues 101–150 was designed to test substitutions at individual positions. To strike a compromise between library size and library diversity, we systemically chose substitutions using two processes. First, at each residue, we substituted alanine along with four other substitutions that were predicted to be less disruptive according to scores from the BLOSUM80 matrix. Five substitutions from the BLOSUM80 matrix were introduced into residues that were originally alanine (Supplementary Table 6). Second, we aligned 38 homologous protein sequences (Supplementary Table 7) from mammalian species with the sequence for human TRBC. Homologous substitutions at a given position that were not already in the library were added.

Where possible, for each substitution, we synthesized two oligos with different codons. As a control, we also tested a single synonymous mutation at each position. Other controls included single-residue deletions (tested at all residues) and stop codons (tested at five individual residues within the mutagenesis region), resulting in a final library size of 649 oligos (Supplementary Table 8). The oligo pool was synthesized (Twist), PCR-amplified and introduced into a plasmid backbone containing a *TRAC* exon-targeted HIT HDRT through Golden Gate assembly.

### HIT site saturation mutagenesis library design and synthesis

gBlocks (IDT) encoding Cβ residues 101–150 were separately synthesized with degenerate nucleotides specified at bases encoding a target residue (G102, D112 or P116). Each gBlock was PCR-amplified and individually introduced into a plasmid backbone containing a *TRAC* exon-targeted HIT HDRT through Golden Gate assembly.

### HIT scanning mutagenesis screen

T cells were edited with *TRAC*-targeted RNP (i1) and AAV6 encoding the pooled HIT library. Edited cells were expanded, immunomagnetically purified with BW242 and sorted on the basis of BW242 binding and HIT expression (using anti-mouse F(ab′)_2_). Bulk RNA was isolated from sorted cells and cells with the original library (Direct-zol RNA microprep kit, Zymo) and used as a template for complementary DNA (cDNA) synthesis (high-capacity cDNA reverse transcription kit, Applied Biosystems).

### HIT site saturation mutagenesis screen

T cells were edited with *TRAC*-targeted RNP (e1) and transduced with AAV6 encoding a single pooled HIT library (with site saturation mutagenesis performed at Cβ residue 102, 112 or 116). Edited cells were expanded and sorted into two bins on the basis of BW242 binding and HIT expression (using anti-mouse F(ab′)_2_). Bulk RNA was isolated from sorted cells and cells with the original library (Direct-zol RNA microprep kit, Zymo) and used as a template for cDNA synthesis (high-capacity cDNA reverse transcription kit, Applied Biosystems).

### Library amplification and analysis

cDNA from sorted and unsorted samples from all screens was processed using a shared workflow. PCR amplification of the library region was performed with Q5 high-fidelity polymerase (NEB) using primers containing Illumina partial adaptors. The resulting amplicons were purified using SPRI beads and submitted for 2× 250-bp paired-end NGS (Amplicon-EZ, GENEWIZ/Azenta).

FASTQ files from NGS were processed using a workflow in Python. Briefly, reads were scanned for conserved sequences upstream and downstream of the library. Reads that contained these sequences (with no permitted mismatches) were trimmed so that only the library region remained, while reads that lacked these sequences were discarded. Trimmed reads were mapped to library members, with no permitted mismatches. Library member abundance within a given sample was calculated as the number of reads mapped to a library member divided by the number of total mapped reads. Reads mapped to designated library controls (stop codons and deletions) were excluded from totals for analysis.

### HIT arrayed library screen

T cells were edited with *TRAC*-targeted RNP (e1) and separately transduced with HDRTs encoding a single HIT variant. After 5 days of expansion, HIT expression, CD3 expression and BW242 binding were assessed by flow cytometry. Edited nontransduced cells were included as a control.

### Target cell culture

CD19^high^ and CD19^low^ firefly luciferase^+^ Nalm6 cell lines were cultured in RPMI supplemented with FBS (10%), sodium pyruvate (1%, Gibco), HEPES buffer (1%, Corning), penicillin–streptomycin (1%), nonessential amino acids (1%) and 2-mercaptoethanol (0.1%, Gibco). CD19 expression in Nalm6 lines was validated by flow cytometry. RFP^+^ A375 cells were cultured in DMEM (Gibco) supplemented with FBS (10%), sodium pyruvate (1%), HEPES buffer (1%) and penicillin–streptomycin (1%).

### CD47 SEED coincubation with NK cells

TCR^−^B2M^−^CD47^+^ cells were generated by performing editing with a *TRAC* exon-targeted RNP (e1), a *B2M* intron-targeted RNP (i4) and a CD47 HDRT. In parallel, TCR^−^B2M^−^CD47^–^ cells were generated by performing editing with a *TRAC* exon-targeted RNP (e1) and a *B2M* exon-targeted RNP (e1). Cells generated with both engineering strategies were immunomagnetically purified with anti-B2M antibody and editing outcomes were assessed by flow cytometry. The two populations were then mixed to achieve an approximate 1:1 ratio of CD47^−^:CD47^+^ cells. To block CD47, T cells were incubated with anti-CD47 antibody (clone: B6H12, BD Biosciences) for 30 min at 37 °C. T cells were cocultured for 5 h with allogeneic activated human NK cells and then the coculture composition was quantified by flow cytometry.

### CD19 cytotoxicity assay

T cells were cocultured with 3 × 10^4^ Nalm6 cells in 96-well flat-bottom plates. T cells were serially diluted (twofold) from an initial 1:1 E:T ratio to a minimum 1:64 E:T ratio and plated in triplicate. Nontreated Nalm6 cells were included as a maximum signal control and Nalm6 cells incubated with Tween-20 (0.2%) were included as a minimum signal control. After a 24-h incubation, d-luciferin (0.75 mg ml^−1^, GoldBio) was added to the plates and luminescence was quantified using a GloMax Explorer microplate reader (Promega). The percentage cytotoxicity was determined as follows: 100% × (1 − (sample − minimum)/(maximum − minimum)).

### NY-ESO-1 cytotoxicity assay

T cells were cocultured with 10^4^ preplated RFP^+^ A375 cells in 96-well flat-bottom plates. T cells were serially diluted (twofold) from an initial 2:1 E:T ratio to a minimum 1:16 E:T ratio and plated in triplicate. The RFP^+^ count per well was quantified every 2 h over a 72-h span using IncuCyte S3 live-cell imaging (Sartorius). A375 cell growth was calculated as the number of RFP^+^ objects at a given time point, normalized to the number of RFP^+^ objects at the start of the assay.

### ddPCR translocation assays

ddPCR assays were designed to measure the occurrence of balanced translocations among TRAC, B2M and CD4. The assays used a pair of primers targeting balanced translocations between two different cut sites and a fluorescent FAM probe (Supplementary Table 3). For all the assays, RPP30 was included as a reference using a primer pair targeting the gene and a fluorescent HEX probe. Therefore, the percentage of the balanced translocation occurrences was calculated as a function of the number of FAM^+^ droplets normalized to the HEX^+^ droplets.

Genomic DNA was purified using the GenFind V3 kit (Beckman) following the manufacturer’s protocol. DNA was quantified using a NanoDrop One (Thermo Fisher Scientific). All DNA samples were digested with HindIII in 10X rCutSmart buffer (NEB) before the ddPCR; 10 U of HindIII was added per microgram of DNA. Samples were then normalized to 100 ng ul^−1^. ddPCR was performed using a QX200 ddPCR System (BioRad) following the manufacturer’s protocols. The reaction mix consisted of ddPCR supermix for probes (no deoxyuridine triphosphate, BioRad), 900 nM of each primer, 450 nM of each probe and 400 ng of purified, digested genomic DNA. A 20-µl PCR reaction was used to generate lipid droplets with an automated Droplet Generator (BioRad). Readout was performed with the QX200 droplet reader (BioRad) and ddPCR droplet reader oil (BioRad). Data analysis was conducted with the QX Manager software (BioRad) and thresholds were set manually to obtain the number of positive droplets for each channel.

### Single-cell RNA sequencing

Cells from different editing conditions were stained with TotalSeq-B Hashtags (Biolegend) according to the manufacturer’s recommendations. An equal number of cells from each condition were pooled. Following the 10X Genomics user guide (CG000417, Rev D), 3 × 10^5^ cells were loaded into four 3′ v3.1 HT reactions of a Chromium Next GEM Chip M on the Chromium X controller for GEM generation. Gene expression and cell surface protein libraries were sequenced at a depth of 20,000 and 5,000 reads per cell, respectively, on four lanes of a 10B NovaSeq X flow cell at the University of California, San Francisco (UCSF) Center for Advanced Technology. FASTQ files were processed with CellRanger version 7.1 and cells with greater than 15% unique molecular identifiers (UMIs) from mitochondrial genes or greater than 200,000 UMIs were removed. Cells were assigned to their condition using hashtag counts with the hashsolo library (scanpy version 1.10.1)^56^. Finally, count matrices were run through the inferCNV R package (version 1.20.0)^57^ pipeline as previously described^48^. Background levels of chromosomal loss were accounted for by subtracting the average frequency of abnormalities observed in the nonedited control condition.

### Data processing and figure creation

Data analysis was performed using Microsoft Excel (version 16.8) and graphs were generated using GraphPad Prism (version 10.1.1). Figures were produced using elements from BioRender.com.

### Reporting summary

Further information on research design is available in the Nature Portfolio Reporting Summary linked to this article.

## Online content

Any methods, additional references, Nature Portfolio reporting summaries, source data, extended data, supplementary information, acknowledgements, peer review information; details of author contributions and competing interests; and statements of data and code availability are available at 10.1038/s41587-024-02531-6.

## Supplementary information

Supplementary Information Supplementary Figs. 1–7.

Reporting Summary

Supplementary Table 1 Supplementary Tables 1–8.

Supplementary File 1 Annotated SEED plasmid maps.

## Source data

Source Data Figs. 1–8 and Extended Data Figs. 2–7, 9 and 10 Statistical source data.

Source Data Fig. 1 Unprocessed gel.

## Data Availability

Sequences for all primers, guides and HDRTs are provided in the Supplementary Information. Additional annotated maps for SEED plasmids are provided in Supplementary File 1. Raw data from single-cell and library sequencing experiments are available from the Sequence Read Archive (PRJNA1187600). Source data are provided with this paper.

## Code availability

Custom code used to analyze HIT mutagenesis libraries is accessible on GitHub (https://github.com/ChrisRChang/SEED-Public-Analysis-Tools.git).
