# Supplementary material for: SEED-Selection enables high-efficiency enrichment of primary T cells edited at multiple loci
Source: Nat Biotechnol. 2025 Feb 5;43(12):2043–53. doi: 10.1038/s41587-024-02531-6 (PMC12320447; doi:10.1038/s41587-024-02531-6)
Supplement: Supplementary file 1 — Supplementary Figs. 1–7. [file 41587_2024_2531_MOESM1_ESM.pdf]

nature biotechnology

Supplementary information https://doi.org/10.1038/s41587-024-02531-6

SEED-Selection enables high-efficiency enrichment of primary T cells edited at multiple loci

In the format provided by the authors and unedited

Contents

Supplementary Tables (external file)**:**

1. SEED yield data
2. HDRT Sequences
3. Primer Sequences
4. gRNA Sequences
5. Antibodies / Dextramers Used
6. Scanning Mutagenesis Substitution Table
7. TRBC Orthologues
8. TRBC Scanning Mutagenesis Oligo Sequences

Supplementary Figures:

1. *TRAC*-CAR single-edit gating strategy
2. *B2M*-CD47 single-edit gating strategy
3. NK co-culture gating strategy
4. HIT^{112K} gating strategy
5. Transgenic TCR gating strategy
6. *CD4*-CD8 single-edit gating strategy
7. *B2M* gDNA PCR unedited gel image and amplicon design

Additional Supplementary Files:

1. SEED_HDRT_designs.gb

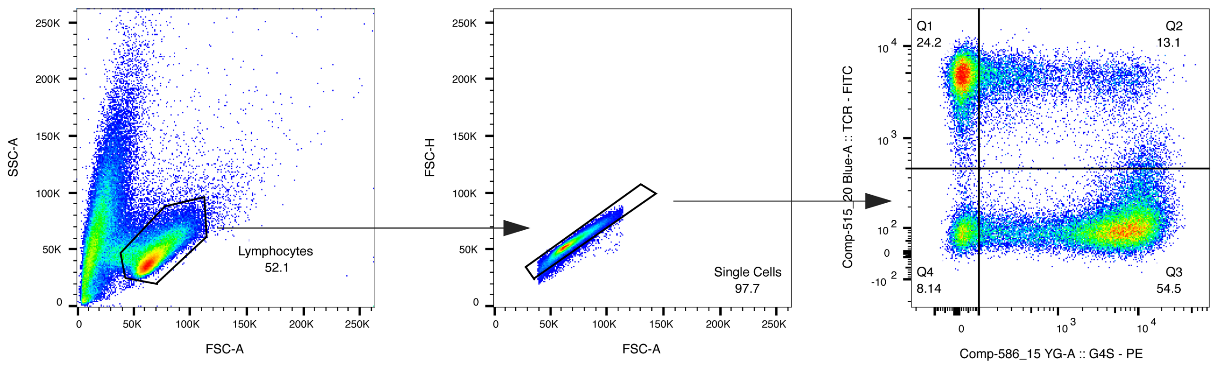

Supplementary Figure 1: General gating strategy for assessing editing outcomes in T cells edited with *TRAC*-targeted HDRTs encoding CARs. Gates for CAR and TCR expression were set based on edited non-transduced controls and non-edited controls.

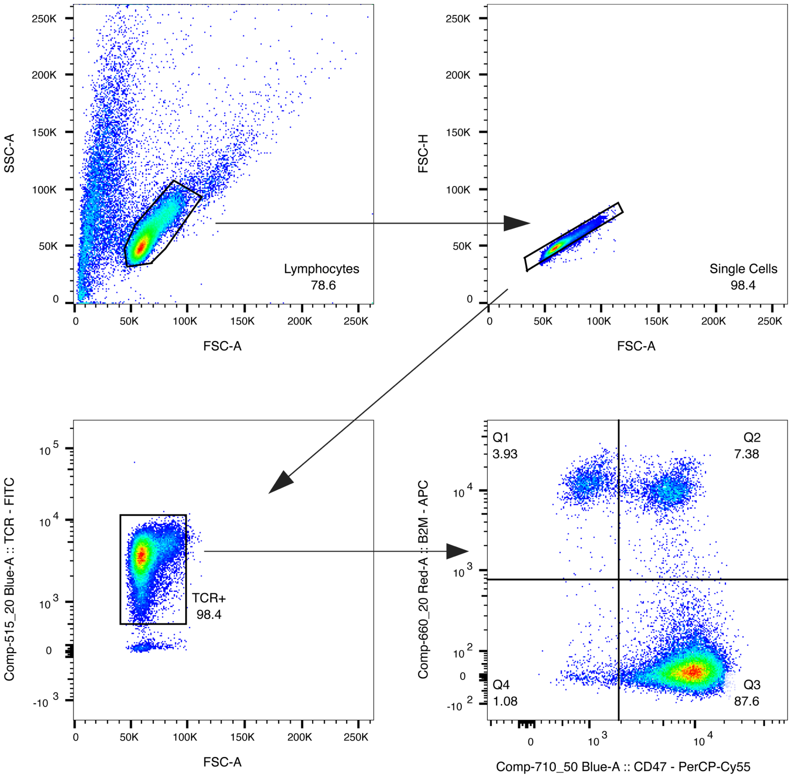

Supplementary Figure 2: Gating strategy for assessing editing outcomes in T cells edited with *B2M*-targeted HDRTs encoding CD47. Gates for B2M and CD47 expression were set based on edited non-transduced controls and unedited controls.

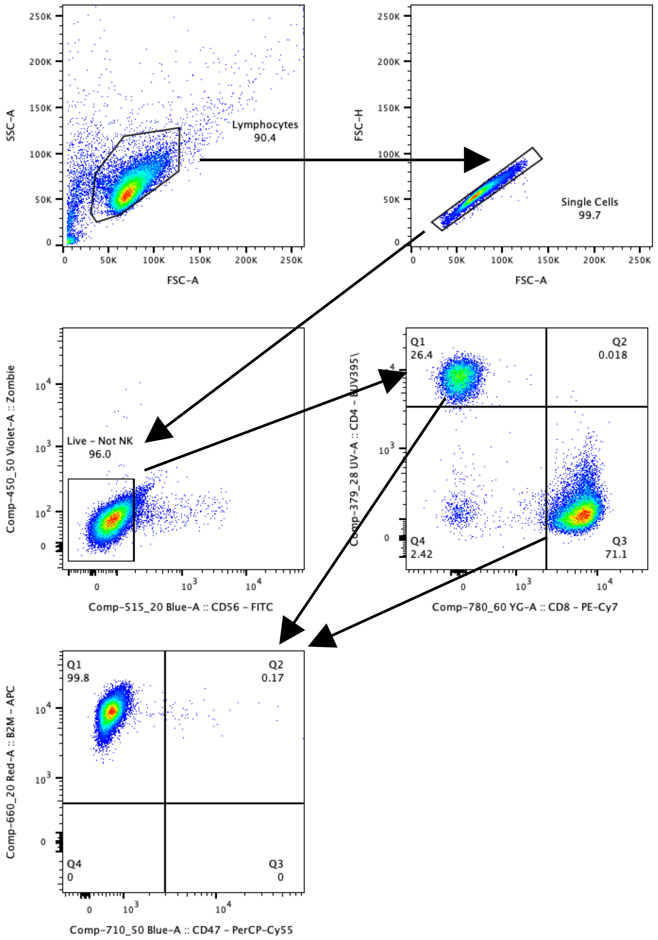

Supplementary Figure 3: Gating strategy for NK cell co-cultures. Gating for CD56 was set based on an NK cell only control sample. T cells were identified through a gate for $CD4^+$ or $CD8^+$ cells.

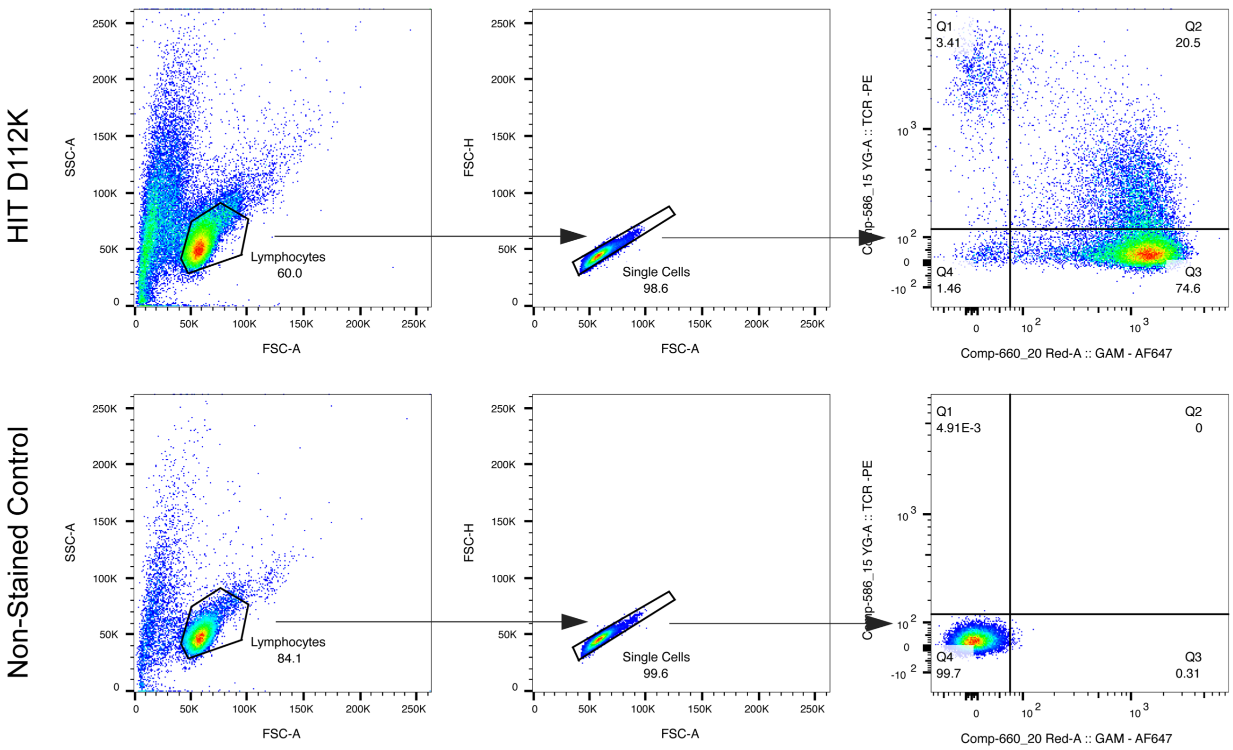

Supplementary Figure 4: Gating strategy for assessing editing outcomes in T cells edited with a *TRAC*-targeted HDRT encoding HIT D112K. Gating for TCR and HIT expression was set based on edited non-transduced samples and non-edited samples.

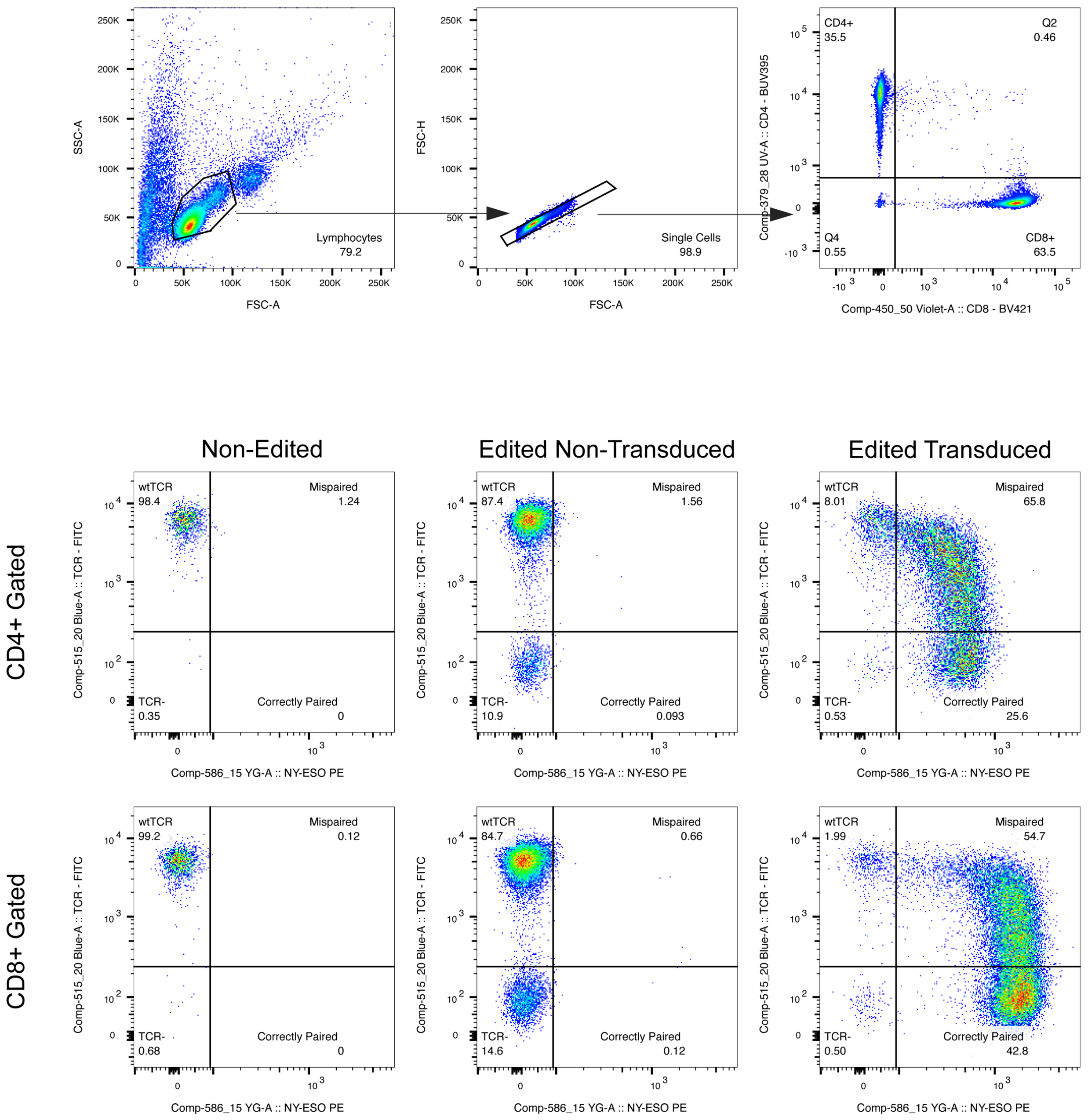

Supplementary Figure 5: General gating strategy for assessing editing outcomes and mispairing in T cells edited with a *TRAC*-targeted HDRT encoding an epitope edited transgenic TCR. Gating for mispairing were set based on edited non-transduced control samples.

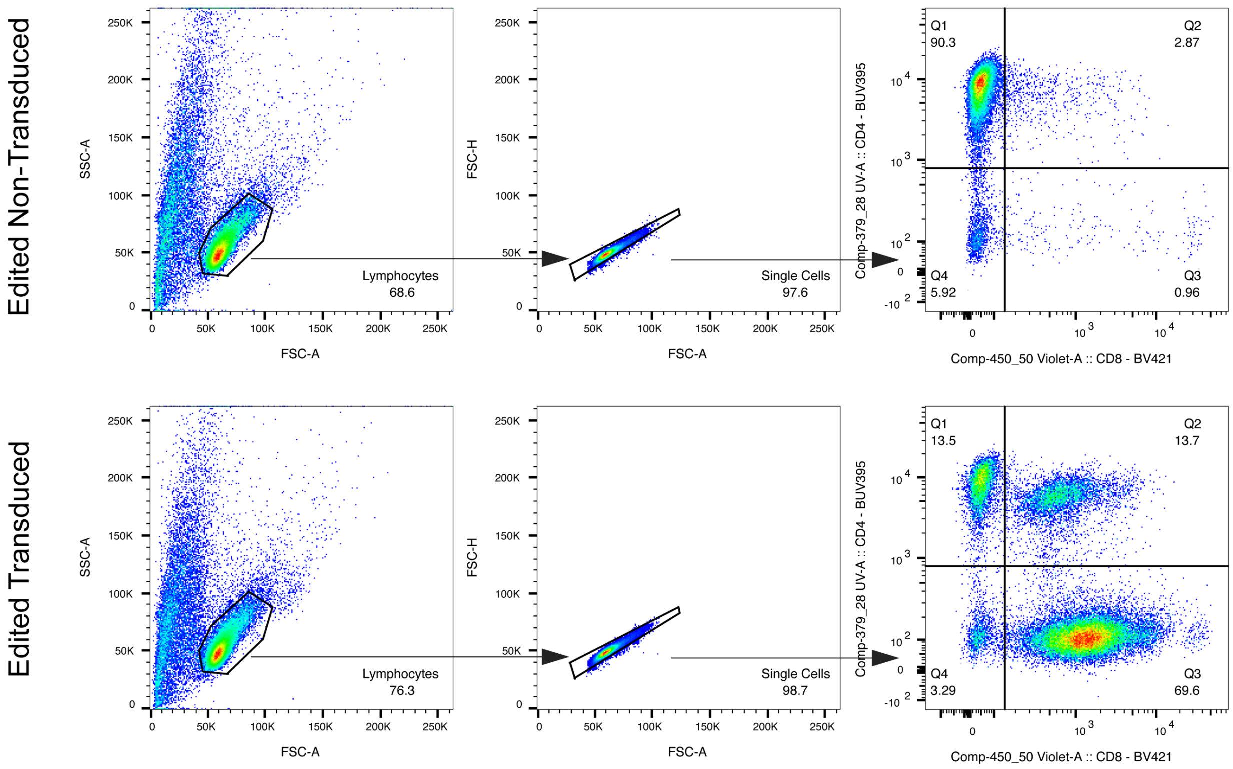

Supplementary Figure 6: Gating strategy for assessing editing outcomes in CD4⁺ T cells edited with a *CD4*-targeted HDRT encoding CD8. Gating for CD4 and CD8 expression was set based on edited non-transduced controls.

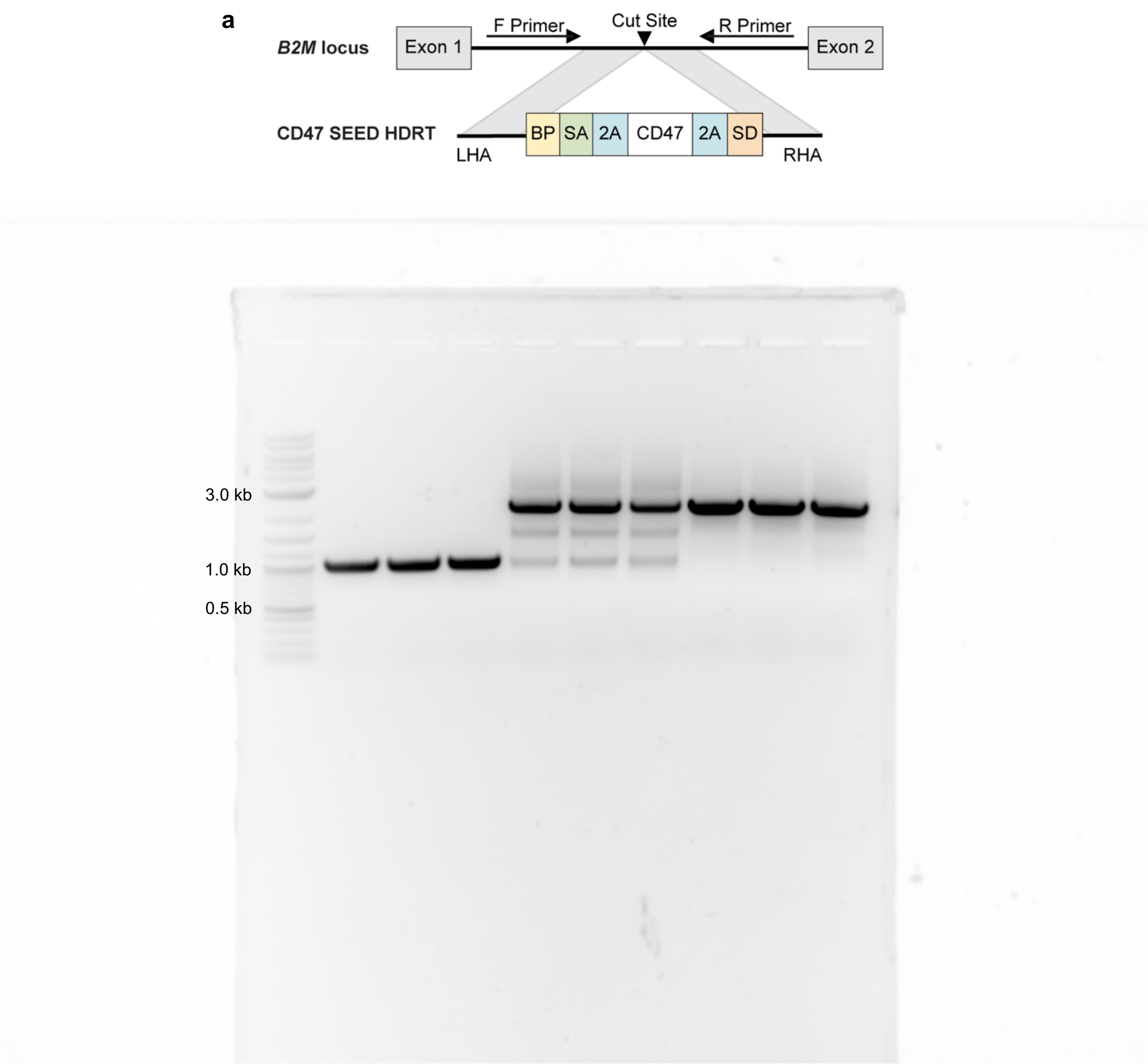

Supplementary Figure 7: a, Primer binding sites for B2M integration PCR. **b,** Unprocessed gel image of *B2M* HDRT integration site gDNA PCR (Cropped gel shown in Fig. 1i).
