# Supplementary material for: SEED-Selection enables high-efficiency enrichment of primary T cells edited at multiple loci
Source: Nat Biotechnol. 2025 Feb 5;43(12):2043–53. doi: 10.1038/s41587-024-02531-6 (PMC12320447; doi:10.1038/s41587-024-02531-6)
Supplement: Supplementary file 2 — Reporting Summary [file 41587_2024_2531_MOESM2_ESM.pdf]

nature portfolio

Corresponding author(s): Justin Eyquem

Last updated by author(s): Dec 2, 2024

# Reporting Summary

Nature Portfolio wishes to improve the reproducibility of the work that we publish. This form provides structure for consistency and transparency in reporting. For further information on Nature Portfolio policies, see our Editorial Policies and the Editorial Policy Checklist.

## Statistics

For all statistical analyses, confirm that the following items are present in the figure legend, table legend, main text, or Methods section.

| n/a | Confirmed | |
|---|---|---|
| ☐ | ☒ | The exact sample size ($n$) for each experimental group/condition, given as a discrete number and unit of measurement |
| ☐ | ☒ | A statement on whether measurements were taken from distinct samples or whether the same sample was measured repeatedly |
| ☐ | ☒ | The statistical test(s) used AND whether they are one- or two-sided<br>*Only common tests should be described solely by name; describe more complex techniques in the Methods section.* |
| ☒ | ☐ | A description of all covariates tested |
| ☒ | ☐ | A description of any assumptions or corrections, such as tests of normality and adjustment for multiple comparisons |
| ☐ | ☒ | A full description of the statistical parameters including central tendency (e.g. means) or other basic estimates (e.g. regression coefficient) AND variation (e.g. standard deviation) or associated estimates of uncertainty (e.g. confidence intervals) |
| ☐ | ☒ | For null hypothesis testing, the test statistic (e.g. $F$, $t$, $r$) with confidence intervals, effect sizes, degrees of freedom and $P$ value noted<br>*Give P values as exact values whenever suitable.* |
| ☒ | ☐ | For Bayesian analysis, information on the choice of priors and Markov chain Monte Carlo settings |
| ☒ | ☐ | For hierarchical and complex designs, identification of the appropriate level for tests and full reporting of outcomes |
| ☒ | ☐ | Estimates of effect sizes (e.g. Cohen's $d$, Pearson's $r$), indicating how they were calculated |

*Our web collection on statistics for biologists contains articles on many of the points above.*

## Software and code

Policy information about availability of computer code

| | |
|---|---|
| Data collection | Insertion and Deletions were determined with the Synthego ICE Analysis (v3) webtool. Live cell imaging data was acquired and analyzed using an Incucyte S3 Live-Cell Analysis System (v2023b). Luminescence data was acquired using a Promega GloMax Explorer (v3.0.0). Readouts for ddPCR were obtained with a QX200 Droplet Reader (BioRad) and ddPCR Droplet Reader Oil (BioRad).<br><br>For single cell RNA sequencing: Gene expression and cell surface protein libraries were sequenced at a depth of 20,000 and 5,000 reads per cell, respectively, on 4 lanes of a 10B NovaSeq X flow cell at the UCSF Center for Advanced Technology. |
| Data analysis | Data analysis was performed using Microsoft Excel (v16.8), and graphs were generated using GraphPad Prism (v10.1.1). Figures were produced using elements from BioRender.com<br><br>FASTQ files from NGS were processed using a workflow in Python (3.8). Briefly, reads were scanned for conserved sequences upstream and downstream of the library. Reads that contained these sequences (with no permitted mismatches) were trimmed so that only the library region remained, while reads that lacked these sequences were discarded. Trimmed reads were mapped to library members, with no permitted mismatches. Library member abundance within a given sample was calculated as: (# of reads mapped to library member) / (# of total mapped reads). Reads mapped to designated library controls (stop codons and deletions) were excluded from totals for analysis. Custom code used to analyze HIT mutagenesis libraries is accessible on GitHub: https://github.com/ChrisRChang/SEED-Public-Analysis-Tools.git.<br><br>Data analysis for ddPCR assays was performed with the QX Manager Software (Bio-Rad), and thresholds were set manually to obtain the number of positive droplets for each channel. |

For single cell RNA sequencing: Fastq files were processed with cellranger (v7.1), and cells with greater than 15% UMIs from mitochndrial genes or greater than 200,000 UMIs were removed. Cells were assigned to their condition using hashtag counts with the hashsolo library (scanpy v1.10.1). Finally, count matrices were run through the inferCNV R package (v1.20.0) pipeline as previously described. Background levels of chromosomal loss were accounted for by subtracting the average frequency of abnormalities observed in the non-edited control condition.

For manuscripts utilizing custom algorithms or software that are central to the research but not yet described in published literature, software must be made available to editors and reviewers. We strongly encourage code deposition in a community repository (e.g. GitHub). See the Nature Portfolio guidelines for submitting code & software for further information.

## Data

Policy information about availability of data

All manuscripts must include a data availability statement. This statement should provide the following information, where applicable:

- Accession codes, unique identifiers, or web links for publicly available datasets
- A description of any restrictions on data availability
- For clinical datasets or third party data, please ensure that the statement adheres to our policy

Values for data plotted in Main and Extended Figures are provided in Source Data File 1. Sequences for all primers, guides, and HDRTs are provided in the supplementary dataset. Additional annotated maps for SEED plasmids are provided in Supplementary File 1. Raw data from single cell and library sequencing experiments are available from the Sequence Read Archive (PRJNA1187600).

## Research involving human participants, their data, or biological material

Policy information about studies with human participants or human data. See also policy information about sex, gender (identity/presentation), and sexual orientation and race, ethnicity and racism.

| | |
|---|---|
| Reporting on sex and gender | The study did not involve human research participants. |
| Reporting on race, ethnicity, or other socially relevant groupings | N/A |
| Population characteristics | N/A |
| Recruitment | N/A |
| Ethics oversight | N/A |

Note that full information on the approval of the study protocol must also be provided in the manuscript.

# Field-specific reporting

Please select the one below that is the best fit for your research. If you are not sure, read the appropriate sections before making your selection.

☒ Life sciences ☐ Behavioural & social sciences ☐ Ecological, evolutionary & environmental sciences

For a reference copy of the document with all sections, see nature.com/documents/nr-reporting-summary-flat.pdf

# Life sciences study design

All studies must disclose on these points even when the disclosure is negative.

| | |
|---|---|
| Sample size | Sample sizes were chosen according to the standards of the field (at least two biological replicates). No statistical method was used to determine sample sizes. |
| Data exclusions | No data were excluded |
| Replication | Replicates were performed as described in the main figure legends, extended data figure legends, and methods. |
| Randomization | Randomization was not performed. Experiments included paired controls for each tested donor. |
| Blinding | Blinding was not performed, as it was not relevant to the types of procedures in this study. |

# Reporting for specific materials, systems and methods

We require information from authors about some types of materials, experimental systems and methods used in many studies. Here, indicate whether each material, system or method listed is relevant to your study. If you are not sure if a list item applies to your research, read the appropriate section before selecting a response.

## Materials & experimental systems

| n/a | Involved in the study |
|---|---|
| ☐ | ☒ Antibodies |
| ☐ | ☒ Eukaryotic cell lines |
| ☒ | ☐ Palaeontology and archaeology |
| ☒ | ☐ Animals and other organisms |
| ☒ | ☐ Clinical data |
| ☒ | ☐ Dual use research of concern |
| ☒ | ☐ Plants |

## Methods

| n/a | Involved in the study |
|---|---|
| ☒ | ☐ ChIP-seq |
| ☐ | ☒ Flow cytometry |
| ☒ | ☐ MRI-based neuroimaging |

# Antibodies

**Antibodies used**

Antibody information is provided in Supplementary Table 5.

**Validation**

BW242/412-PE and BW242/412-FITC were validated by manufacturer Miltenyi Biotec for human cell flow cytometry. Specificity was confirmed by confirmed by comparing clones to a relevant reference clone of the same specificity. BW242/412-Biotin was validated by manufacturer Miltenyi Biotec for immunomagentic depletion of Human TCR+ cells.

IP26-BV421, SK3-FITC, OKT4-PE-Cy7, 2M2-APC, UCHT1-BV711, 2M2-Biotin, and SK3-Biotin were validated by manufacturer Biolegend for human cell flow cytometry.

SK3-BUV395, SK1-BV421, SK1-PE-Cy7, B6H12-PerCP-Cy5.5, and SJ25C1-BUV373 were validated by manufacturer BD Biosciences for human cell flow cytometry.

E7O2V-PE was tested by manufacturer Cell Signaling for human cell flow cytometry.

Anti-Mouse F(ab')2-AF647 was validated for specificity and cross-reactivity with human proteins by manufacturer Jackson ImmunoResearch.

SLLMWITQV-Dextramer and ELAGIGILTV-Dextramer were validated for by manufacturer Immudex for human cell flow cytometry.

# Eukaryotic cell lines

Policy information about cell lines and Sex and Gender in Research

**Cell line source(s)**

Primary adult blood cells from anonymous healthy human donors were purchased as leukapheresis packs (Stemcell). Frozen primary human NK cells were purchased from Stemcell. Nalm6 CD19 High and CD19 Low cell lines were generated in the Eyquem Lab. A375 cells were recieved from the lab of Julia Carnevale. HEK293T cells were purchased from ATCC (#CRL-3216)

**Authentication**

CD19 expression in Nalm6 lines was validated via flow cytometry prior to functional assays. No authentication was performed for A375 cells

**Mycoplasma contamination**

Primary T and NK cells were not screened for mycoplasma contamination. Nalm6, A375, and 293T lines tested negative for mycoplasma contamination.

**Commonly misidentified lines**
(See ICLAC register)

No commonly misidentified cell lines were used.

# Plants

**Seed stocks**

N/A

**Novel plant genotypes**

N/A

**Authentication**

N/A

## Flow Cytometry

### Plots

Confirm that:

☒ The axis labels state the marker and fluorochrome used (e.g. CD4-FITC).

☒ The axis scales are clearly visible. Include numbers along axes only for bottom left plot of group (a 'group' is an analysis of identical markers).

☒ All plots are contour plots with outliers or pseudocolor plots.

☒ A numerical value for number of cells or percentage (with statistics) is provided.

### Methodology

| | |
|---|---|
| Sample preparation | Cells were resuspended in FACS buffer (phosphate-buffered saline (PBS), 2% FBS, and 1 mM EDTA) and stained with antibodies/dextramer (Supplementary Table 5). Zombie Violet (BioLegend) or Ghost Dye Red (Tonbo) were used in experiments where viability was assessed via flow cytometry. In experiments where HIT or CAR expression was assessed, cells were initially stained with anti-mouse F(ab')2 and then blocked with mouse serum (MilliporeSigma) before antibody staining was performed. In experiments where anti-B2M and MHC-I dextramers were both used, cells were stained with antibodies first, washed, and then stained with dextramer. |
| Instrument | Flow cytometry was performed on a BD FACSymphony Fortessa X-50 or an Attune NxT. Cell sorting was performed on a BD FACSAria. |
| Software | Flow cytometry data was analyzed with FlowJo v10. |
| Cell population abundance | Post-sort purity was not reassessed. |
| Gating strategy | Lymphocytes were gated based on FSC-A vs SSC-A. Single cells were then gated based on FCS-H vs FSC-A. Gates were set based on unedited control samples, edited non-transduced control samples, and non-stained control samples. |

☒ Tick this box to confirm that a figure exemplifying the gating strategy is provided in the Supplementary Information.
