# Supplementary figures and images for: SEED-Selection enables high-efficiency enrichment of primary T cells edited at multiple loci
Source: Nat Biotechnol. 2025 Feb 5;43(12):2043–53. doi: 10.1038/s41587-024-02531-6 (PMC12320447; doi:10.1038/s41587-024-02531-6)

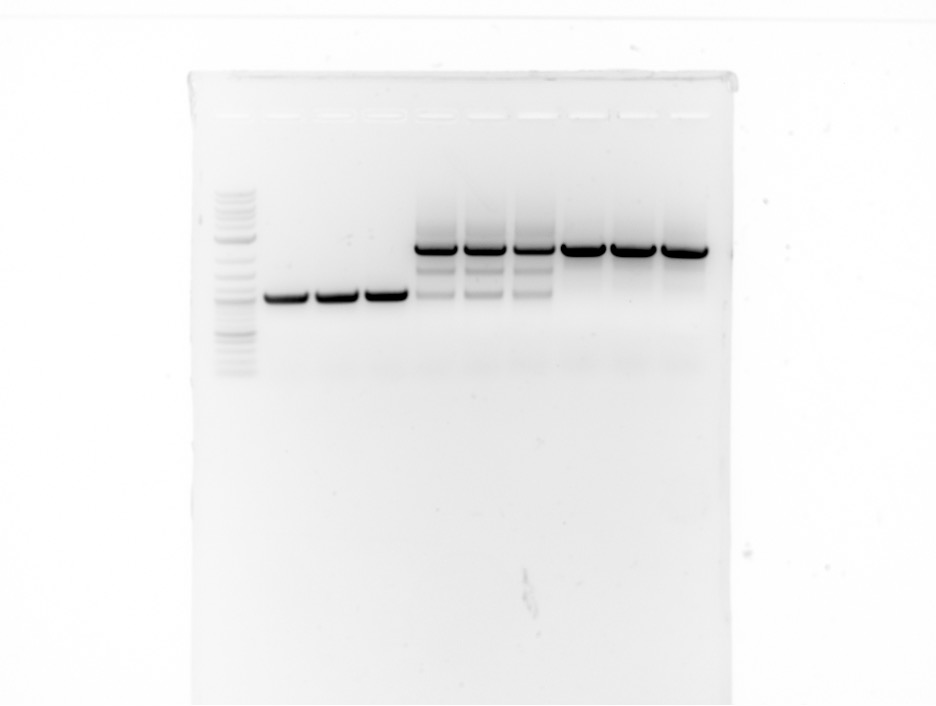

Supplement: Supplementary file 6 — Unprocessed gel. [file 41587_2024_2531_MOESM6_ESM.jpg]
